# Biologically inspired bioactive hydrogels for scarless corneal repair

**DOI:** 10.1126/sciadv.adt1643

**Published:** 2024-12-18

**Authors:** Jianan Huang, Tuoying Jiang, Jiqiao Qie, Xiaoyu Cheng, Yiyao Wang, Yang Ye, Zhuoheng Yang, Hongji Yan, Ke Yao, Haijie Han

**Affiliations:** ^1^ Eye Center, the Second Affiliated Hospital, School of Medicine, Zhejiang University, Zhejiang Provincial Key Laboratory of Ophthalmology, Zhejiang Provincial Clinical Research Center for Eye Diseases, Zhejiang Provincial Engineering Institute on Eye Diseases, Hangzhou 310009, P. R. China.; ^2^ MOE Laboratory of Biosystems Homeostasis and Protection and College of Life Sciences-iCell Biotechnology Regenerative Biomedicine Laboratory, College of Life Sciences, Zhejiang University, Hangzhou 310058, P. R. China.; ^3^ Science for Life Laboratory, Division of Nanobiotechnology, Department of Protein Science, Royal Institute of Technology (KTH), 171 65, Solna, Sweden.; ^4^ Department of Medical Cell Biology, Uppsala University, 752 36 Uppsala, Sweden; and AIMES – Center for the Advancement of Integrated Medical and Engineering Sciences at Karolinska Institutet and KTH Royal Institute of Technology, 171 65 Stockholm, Sweden.; ^5^ State Key Laboratory of Trauma Burn and Combined Injury, Third Military Medical University, Chongqing 400038, P. R. China.

## Abstract

Corneal injury–induced fibrosis occurs because of corneal epithelial basement membrane (EBM) injury and defective regeneration. Corneal fibrosis inhibition and transparency restoration depend on reestablished EBM, where the collagen network provides structural stability and heparan sulfate binds corneal epithelium–derived cytokines to regulate homeostasis. Inspired by this, bioactive hydrogels (Hep@Gel) composed of collagen-derived gelatins and highly anionic heparin were constructed for scarless corneal repair. Hep@Gel resembled the barrier function of the EBM regarding surface-confined binding, long-time sequestration, and progressive degradation of IL-1, TGF-β, and PDGF-BB, which robustly inhibited the apoptosis and myofibroblast transition of keratocytes. Animal models of rabbits and nonhuman primates confirmed that Hep@Gel effectively limited the influx of inflammatory and fibrotic cytokines from the epithelium into the stroma to down-regulate the wound healing cascade, contributing to better vision quality with 73% reduced fibrosis. Hep@Gel offers a solution for preventing corneal injury–induced scarring and substituting for lamellar keratoplasty to remove scarring.

## Teaser

Corneal epithelial basement membrane–inspired hydrogel provides a potential solution for corneal scarring–induced blindness.

## INTRODUCTION

Corneal scarring manifesting as opacity is a common pathological process occurring upon injury, which may eventually progress to corneal blindness (*1*–*4*). It is estimated that more than 12 million people are suffering from corneal scarring–induced blindness worldwide, making corneal blindness the second leading cause of blindness in most developing countries (*5*, *6*). Now, therapies for corneal scarring include the use of steroid drugs to prevent scarring and lamellar keratoplasty to remove scarring tissues (*7*, *8*), which are accompanied by high risks of steroid-induced complications or the extreme shortage of donor corneas, respectively. So far, more than 98.5% of patients await a donor cornea, with one cornea available for every 70 needed (*9*). There is an unmet demand to develop a therapeutic modality to treat corneal injury–induced scarring.

Corneal scarring is formed because of the unrestrained wound healing cascade (*10*), involved with the disordered stromal homeostasis full of inflammatory and fibrotic factors released from the injured epithelium (*11*). At the early stage of the wound healing cascade, inflammatory cytokines like interleukin-1 (IL-1) stimulate the quiescent keratocytes to undergo apoptosis or transformation into fibroblasts. Besides, immune cells are recruited to trigger the inflammatory cascade. At a later stage, fibroblasts are further induced to myofibroblasts with a sufficient influx of fibrotic factors including transforming growth factor–β (TGF-β) and platelet-derived growth factor BB (PDGF-BB), contributing to corneal opacity (*11*, *12*). Encouraged by the booming advances in the fields of biomaterials (*13*), researchers have investigated a variety of hydrogels for corneal repair (*9*, *14*–*17*). For instance, gelatin-based hydrogel or decellularized corneal extracellular matrix (ECM)–based hydrogel could seal corneal defects, replenish ECM, and promote wound healing (*18*–*20*). However, corneal scarring has not been well addressed in these studies. Accordingly, stem cells, exosomes, or drugs are loaded into hydrogels to modulate the wound microenvironment and suppress scarring (*15*, *16*, *21*–*23*). Despite the positive outcomes, it will inevitably encounter a series of problems. The in vivo safety evaluation of stem cells should be extensively examined (*24*), and the survival of transplanted cells is usually poor due to the deteriorative microenvironment (*25*). Besides, batch-to-batch variation exists for cells and exosomes while drugs may have unexpected off-target effects. Consequently, cell-free, exosome-free, and drug-free strategies are greatly needed to attenuate corneal scarring effectively.

The corneal epithelial basement membrane (EBM), located at the interface between epithelium and stroma, separates these two layers spatially and limits the passage of epithelium-derived cytokines into the stroma, which is essential for corneal homeostasis (*26*, *27*). The EBM’s molecular components mainly comprise collagens, heparan sulfate proteoglycans (HSPGs), laminins, and nidogens (*28*). Specifically, the sequestration of cytokines within the EBM is mediated by the electrostatic interactions between the positively charged residues of the cytokines and the substantial sulfate anion of heparin (*27*, *29*, *30*). Upon corneal epithelial-stromal injury that disrupts the EBM, the influx of abundant epithelium-derived cytokines contributes to the amplified wound healing cascade, breaking the stromal homeostasis and progressing to corneal scarring (*31*). Therefore, restoring the EBM seems to be a highly efficient strategy to restrain the wound healing cascade.

**Fig. 1.**
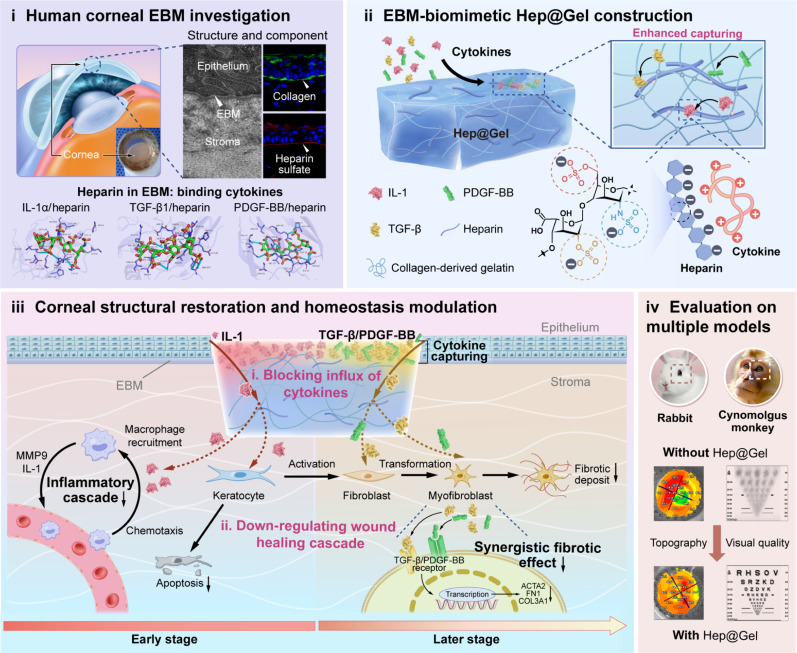
Human EBM-inspired bioactive Hep@Gel for scarless corneal wound healing. EBM-inspired Hep@Gel captures inflammatory and fibrotic cytokines, blocking the passage of cytokines from the injured epithelium to the stroma, which markedly down-regulates the wound healing cascade to coordinate the stromal homeostasis and promotes scarless corneal repair of both rabbits and cynomolgus monkeys.

Herein, we propose the “biomimetic EBM” strategy as a potential solution for corneal scarring (Fig. 1), with bioactive Hep@Gel bioengineered from methacrylated gelatin (GelMA) and thiolated heparin (HepSH), which could robustly modulate the microenvironment without additional cells or drugs. First, Hep@Gel was examined for its transparency, mechanical strength, and adhesive properties. Then, the cytokine-capturing ability of the hydrogel was investigated. Because of the robust cytokine binding capability of Hep@Gel, long-time sequestration and progressive degradation of IL-1, TGF-β, and PDGF-BB were observed. Notably, the bound cytokines were predominantly confined to the hydrogel surface, rendering that Hep@Gel highly resembles the native EBM.Encouraged by the barrier function of Hep@Gel presented in the in vitro corneal injury model, the hydrogel’s therapeutic effect was further extensively examined in rabbit and nonhuman primate models of corneal injury. Hep@Gel could limit the influx of cytokines and ultimately down-regulate the wound healing cascade, contributing to organized corneal repair. Considering that the animal models simulate the conditions of both corneal injury and post-corneal debridement, it was concluded that Hep@Gel could potently prevent scarring or substitute for lamellar keratoplasty to remove scarring tissues. This EBM-inspired bioactive hydrogel may offer a pleiotropic strategy for treating corneal scarring–related blindness.

## RESULTS

### Investigation of human corneal EBM

The corneal EBM plays a central role in maintaining homeostasis, which is crucial for corneal transparency (*1*). Thereby, the structure and composition of the human cornea, especially the EBM, were carefully investigated to inspire the potential solutions for corneal scarring.

**Fig. 2.**
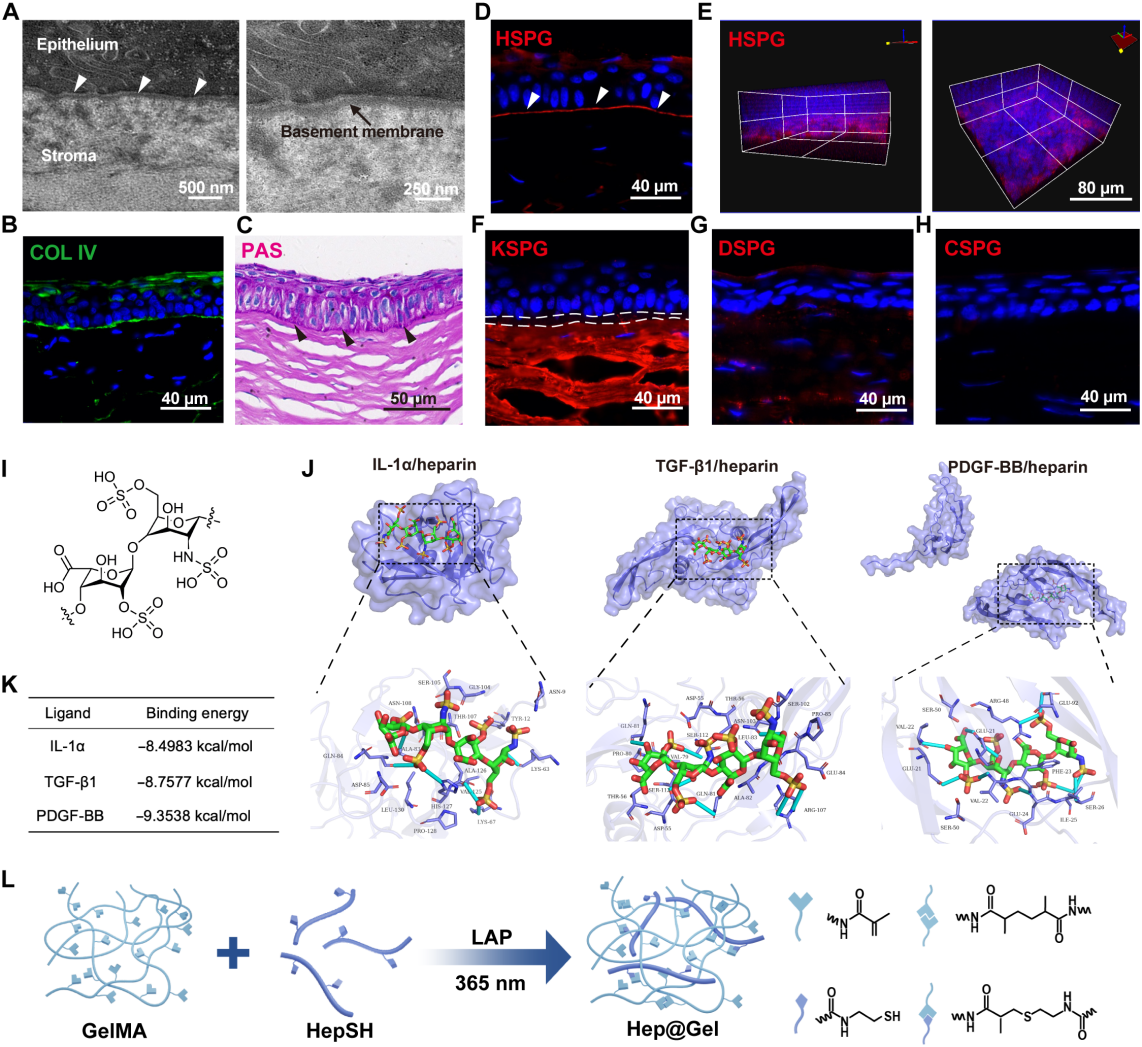
Human corneal EBM-inspired design of Hep@Gel. (**A**) Transmission electron microscopy (TEM) images of the human corneal EBM. White arrowheads indicated the EBM. (**B**) Immunostaining of COL IV in the cornea. (**C**) Periodic acid–Schiff (PAS) staining to detect the polysaccharides in the cornea. Black arrowheads indicated the polysaccharides in the EBM. (**D** and **E**) Immunostaining of HSPG in the cornea. White arrowheads indicated HSPG in the EBM. (**F** to **H**) Immunostaining of keratan sulfate proteoglycan (KSPG), dermatan sulfate proteoglycan (DSPG), and chondroitin sulfate proteoglycan (CSPG) in the cornea. White dashed lines in (F) depicted the negative expression of KSPG in the EBM. (**I**) Structure of heparin. (**J**) Results of computational docking analysis between various cytokines and heparin using MOE software. (**K**) The binding energy between various cytokines and heparin. (**L**) The design and preparation of Hep@Gel.

Transmission electron microscopy (TEM) images confirmed the existence of the EBM positioned between basal epithelial cells and the stroma (Fig. 2A), which was mainly composed of collagen IV (Fig. 2B), consistent with previous reports that collagen IV forms the network of the EBM (*26*). Besides, periodic acid–Schiff (PAS) staining depicted the abundant polysaccharides along the EBM (Fig. 2C), which are the major components of proteoglycans. There are altogether four kinds of proteoglycans presented in natural ECM (*32*), including HSPG, keratan sulfate proteoglycan (KSPG), dermatan sulfate proteoglycan (DSPG), and chondroitin sulfate proteoglycan (CSPG); thus, we exhaustively examined all these proteoglycans in the EBM (Fig. 2, D to H). While KSPG was highly expressed in the underlying stromal layer, HSPG was the major proteoglycan in the EBM, which was known to contain abundant heparan sulfate. As natural polysaccharides, both heparan sulfate and heparin capture multiple cytokines, a process mediated by electrostatic interactions between positively charged amino acid residues of cytokines and negatively charged sulfate groups of heparan sulfate and heparin (*30*). Notably, heparin, a highly sulfated form of heparan sulfate, carries a higher anionic charge density (*33*), which is thought to regulate the local concentration of cytokines in the microenvironment vigorously (Fig. 2I). Therefore, we focused on heparin and further analyzed the capability of heparin to capture corneal injury–related inflammatory and fibrotic cytokines. The results of computational docking ensured the high binding affinity of IL-1, TGF-β, and PDGF-BB to heparin, with a binding energy of −8.4983, −8.7577, and −9.3538 kcal/mol, respectively (Fig. 2, J and K). Inspired by the components of the EBM and the robust cytokine-capturing ability of heparin, collagen-derived gelatins together with proteoglycan-related heparin were used to develop the bioactive Hep@Gel to simulate the corneal EBM and coordinate homeostasis upon corneal injury (Fig. 2L).

### Preparation and characterization of Hep@Gel

Hep@Gel was composed of two main components simulating the corneal EBM: collagen-derived gelatins and heparin, which in detail is prepared using methacrylate-modified gelatin GelMA and sulfhydryl-modified heparin HepSH, followed by photocrosslinking (figs. S1 to S3). As expected, GelMA and HepSH could be photocured using lithium phenyl (2,4,6-trimethylbenzoyl) phosphinate (LAP) as the photoinitiator to obtain Hep@Gel, and the gel time decreased as the solid contents rose (figs. S4 and S5). While the Hep@Gel precursor solution with GelMA (300 mg/ml) had poor fluidity and gelled too quickly within 5 s, it was quite inconvenient in clinical practice. Therefore, Hep@Gel containing GelMA (100 or 200 mg/ml) was further examined.

Because transparency is vital for the corneal refractive effect, we explored the transmittance of Hep@Gel (fig. S6). In the wavelength range of visible light (400 to 800 nm), Hep@Gel showed good transparency with a transmittance greater than 80% except for Hep20@Gel^100^ [Hep@Gel with HepSH (20 mg/ml) and GelMA (100 mg/ml)] and Hep20@Gel^200^. These two hydrogels with compromised transparency might be attributed to the relatively high proportion of HepSH in the solid contents, and they were not adopted for further investigation.

**Fig. 3.**
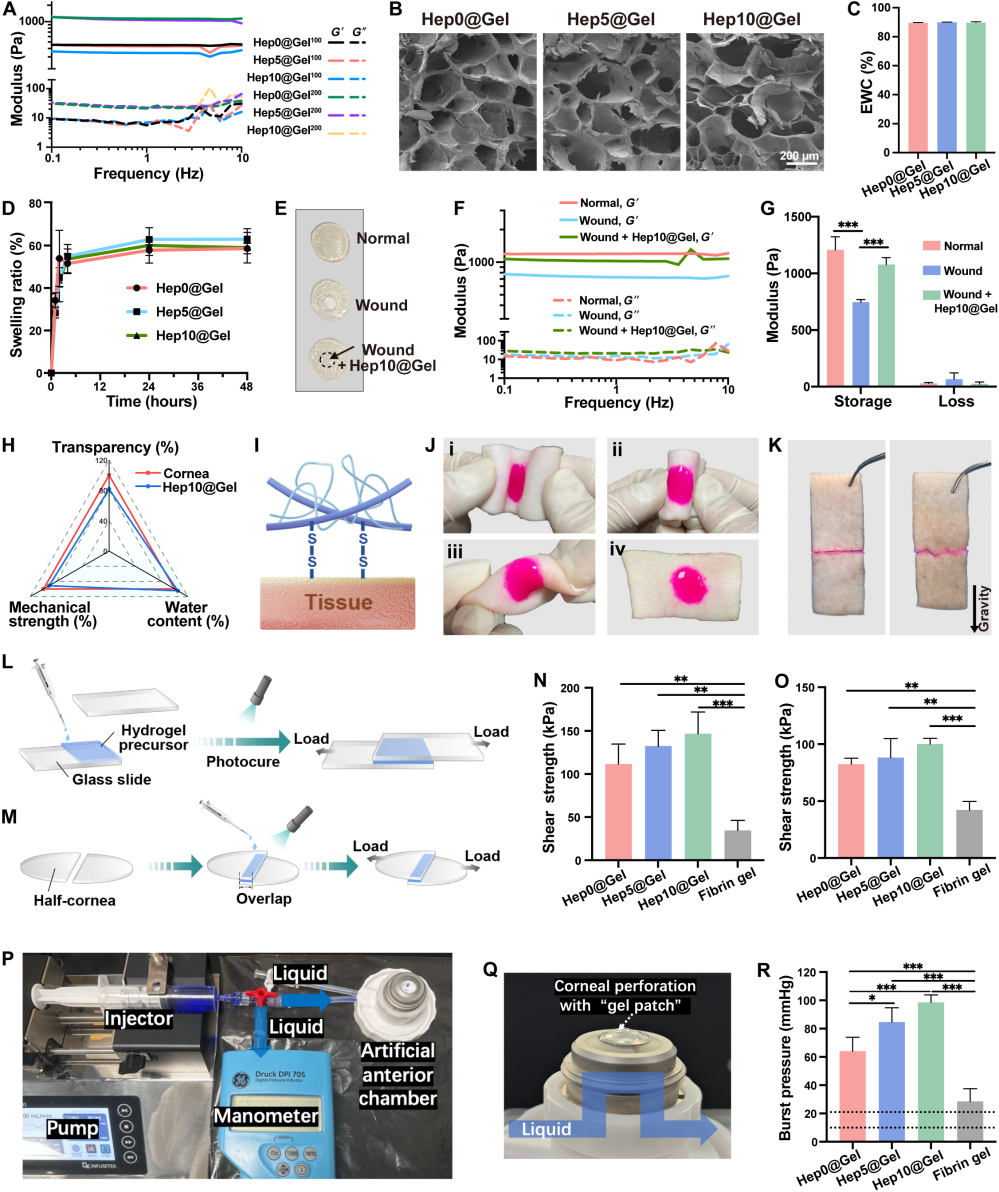
Characterization of Hep@Gel. (**A**) Mechanical properties of Hep@Gel. *G*′, storage modulus. *G*″, loss modulus. Hep0@Gel^100^ referred to the hydrogels with HepSH (0 mg/ml) and GelMA (100 mg/ml). The other hydrogels were similarly defined (*n* = 3). (**B**) Scanning electron microscopy (SEM) images of Hep@Gel. Here and thereafter, Hep0@Gel, Hep5@Gel, and Hep10@Gel referred to Hep0@Gel^200^, Hep5@Gel^200^, and Hep10@Gel^200^, respectively. (**C** and **D**) Equilibrium water content (EWC) and mass swelling ratios of Hep@Gel (*n* = 3). (**E** and **F**) Representative images and storage/loss moduli of normal and wounded cornea buttons as well as wounded cornea buttons with the repair of Hep10@Gel (*n* = 3). The dashed circle in (E) indicated Hep10@Gel. (**G**) Hep10@Gel increased the storage moduli of wounded cornea buttons [*n* = 3, two-way analysis of variance (ANOVA) test]. (**H**) Comparison between the native cornea and Hep10@Gel. (**I**) Disulfide bond–based adhesive mechanism of Hep@Gel. (**J**) Hep10@Gel (stained by rhodamine B) adhered to porcine skin under folding and torsion. (**K**) Adhesion of porcine skin by Hep10@Gel. (**L** and **M**) Schematic illustration of the lap-shear tests using glass slides and porcine corneas. (**N**) The shear strength of Hep@Gel or fibrin glue on glass slides and (**O**) corneas (*n* = 3, one-way ANOVA test). (**P** and **Q**) Schematic illustration of the burst pressure measurement. (**R**) Intraocular burst pressure in ex vivo models of corneal perforation (*n* = 4, one-way ANOVA test). *P < 0.05, **P < 0.01, and ***P < 0.001.

The mechanical strength of Hep@Gel turned out to be tunable (Fig. 3A). The moduli of Hep@Gel with GelMA of 200 mg/ml were superior to those of Hep@Gel with GelMA of 100 mg/ml, and the mechanical strength of Hep@Gel with GelMA of 200 mg/ml was close to each other. Considering the compromised mechanical strength of the wounded cornea, Hep@Gel containing GelMA of 200 mg/ml should be advantageous for corneal repair. Herein and thereafter, Hep0@Gel, Hep5@Gel, and Hep10@Gel referred to Hep0@Gel^200^, Hep0@Gel^200^, and Hep0@Gel^200^, respectively. The microstructure of Hep@Gel revealed by scanning electron microscopy (SEM) indicated that the skeleton of Hep0@Gel, Hep5@Gel, and Hep10@Gel was similar (Fig. 3B), accounting for the analogous mechanical strength among them. The equilibrium water content (EWC) of Hep0@Gel, Hep5@Gel, and Hep10@Gel was near 90% (Fig. 3C), resembling that of the native cornea (85.7 ± 1.5%) (*20*), which provided a mild reparative microenvironment. Besides, the swelling ratios of Hep0@Gel, Hep5@Gel, and Hep10@Gel in phosphate-buffered saline (PBS) reached a maximum of ~60% in 24 hours (Fig. 3D), lower than that of previously reported hydrogels for corneal repair (*34*, *35*), revealing the shape and structural stability of Hep@Gel to ensure proper docking between the hydrogel and the corneal defect.

We further examined whether Hep@Gel could rescue the compromised mechanical strength of the wounded cornea. We applied Hep10@Gel to the defective area of the wounded cornea (Fig. 3E), and it improved the storage modulus of the wounded cornea to the normal one (Fig. 3, F and G), implying the proper integration between Hep10@Gel and the wounded cornea. Collectively, considering the properties of transparency, mechanical strength, and water content, Hep10@Gel was similar to the native cornea (Fig. 3H), suggesting the potential application for corneal repair.

### In vitro and ex vivo adhesive properties of Hep@Gel

Generally, the tissue-binding ability of hydrogels helps to avoid detachment from target tissues and contributes to proper biointegration between hydrogels and adjacent tissues. Herein, Hep@Gel was supposed to have robust adhesion strength due to the disulfide bond–based adhesive mechanism (Fig. 3I). Hep@Gel could adhere tightly to the porcine skin without obvious breakage or detachment, regardless of all deformations (Fig. 3J). Hep@Gel also directly stuck two pieces of porcine skin together (Fig. 3K). Further, to quantitatively investigate the adhesion performance of Hep@Gel, the shear strength of hydrogels on glass slides and porcine corneas was measured successively (Fig. 3, L and M). As the content of HepSH increased from 0 to 10 mg/ml, the shear strength of Hep@Gel rose stepwise from 111.6 ± 23.1 to 146.7 ± 25.2 kPa for glass slides and from 82.4 ± 5.2 to 100.0 ± 5.1 kPa for porcine corneas, respectively (Fig. 3, N and O). Notably, the shear strength of all Hep@Gel was higher than that of the clinically available sealant fibrin gel.

Moreover, the ex vivo adhesion performance of Hep@Gel on the porcine corneas was measured with the burst pressure test. Accordingly, a 3-mm corneal perforation was created, and Hep@Gel or fibrin glue was used to seal the perforation (Fig. 3, P and Q, and movie S1). The results indicated that the burst pressures of all tested Hep@Gel adhesive hydrogels (HepSH, 0 to 10 mg/ml) were higher than that of fibrin glue, ranging from 64.0 ± 9.9 to 98.5 ± 5.3 mmHg for Hep@Gel as compared to 28.5 ± 9.0 mmHg for fibrin glue (Fig. 3R). Considering that the normal intraocular pressure ranges from 10 to 21 mmHg, the burst pressure of Hep10@Gel was approximately six times higher than the normal intraocular pressure, ensuring the firm adhesion of Hep10@Gel to the cornea. Conclusively, the adhesive hydrogels engineered with HepSH (10 mg/ml) and GelMA (200 mg/ml; Hep10@Gel) showed the optimal adhesive ability and the highest burst pressure resistance, which might serve as a promising adhesive biomaterial for corneal repair.

### Enhanced capturing and reduced diffusion of cytokines in Hep@Gel

The intact EBM usually sequesters a few cytokines secreted by corneal epithelial cells. Upon corneal injury that disrupts the EBM, sharp and substantial inflammatory and fibrotic cytokines flood into the stroma, amplifying the wound healing cascade, eventually resulting in fibrosis (*11*, *36*). Thereby, the cytokine-capturing ability of bioactive Hep@Gel was essential for restoring the barrier function of the EBM, which was investigated systematically herein.

**Fig. 4.**
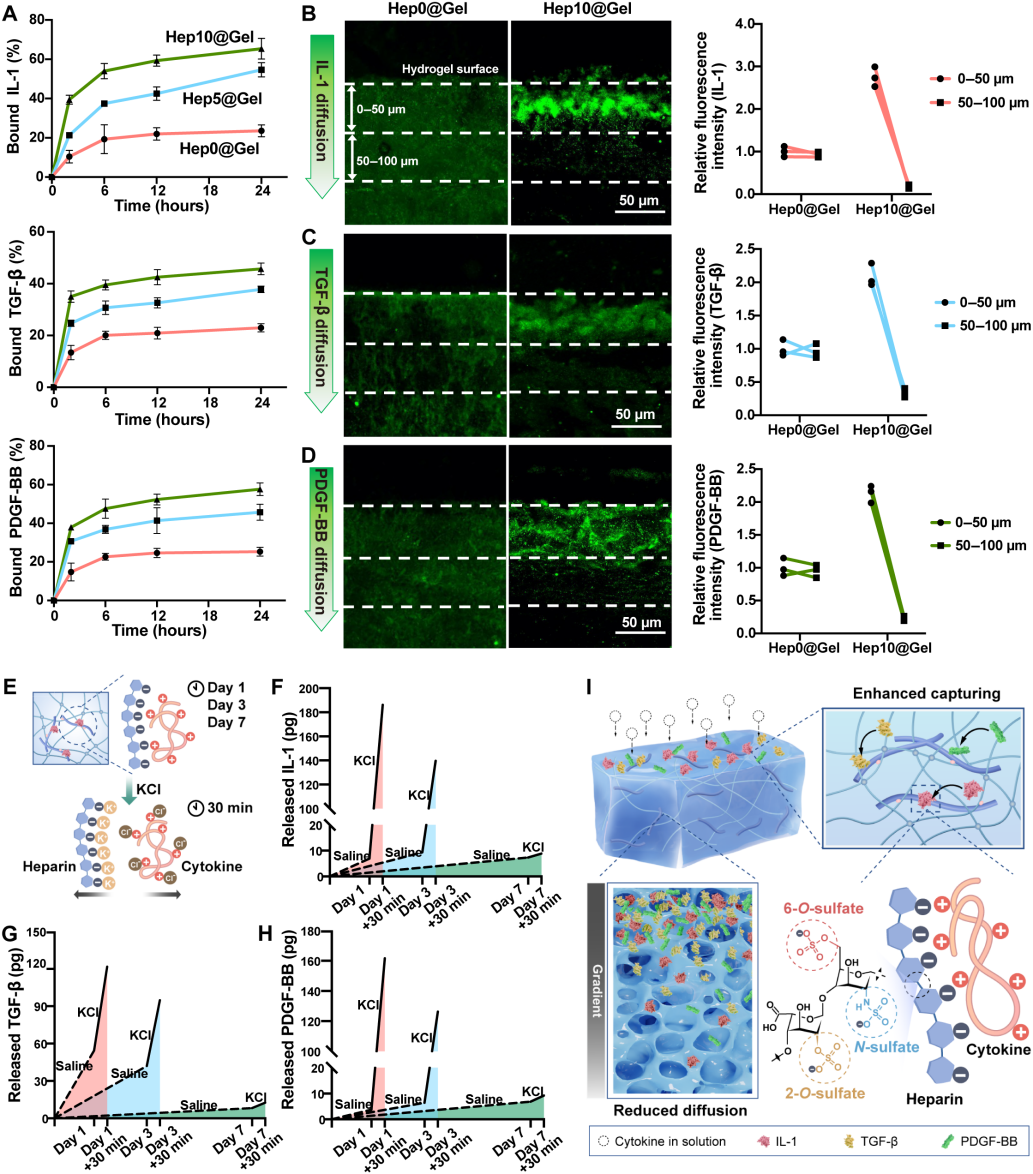
Cytokine capturing ability of Hep@Gel upon exposure to inflammatory and fibrotic cytokines. (**A**) Quantification of the cytokine capturing ability of various Hep@Gel hydrogels (*n* = 3). (**B** to **D**) Detection of the cytokine diffusion in Hep0@Gel and Hep10@Gel. Cytokine diffusion was quantified on the basis of the fluorescence intensity of areas from 0- to 50-μm depth and from 50- to 100-μm depth (*n* = 3). (**E**) Scheme of characterization of the cytokine sequestration in Hep10@Gel. Cytokine-bound hydrogels were first immersed in normal saline to detect the spontaneous release of bound cytokines. KCl was then used to destroy the electrostatic interactions via the shielding effect to determine the remaining bound cytokines. (**F** to **H**) The cytokines were released from Hep10@Gel on days 1, 3, and 7 to evaluate the long-time sequestration and degradation of cytokines in Hep10@Gel (*n* = 3). (**I**) Schematic illustration of the enhanced capturing and reduced diffusion of cytokines in Hep@Gel.

First, it was confirmed that injured human corneal epithelial cells (HCECs) secreted substantial inflammatory cytokines (fig. S7). Inspired by the computational docking analysis (Fig. 2K), Hep@Gel was supposed to vigorously capture IL-1, TGF-β, and PDGF-BB.Therefore, the binding kinetics of these cytokines to Hep@Gel was investigated (Fig. 4A). Unexpectedly, 39.4% of IL-1, 35.0% of TGF-β, and 37.9% of PDGF-BB were sequestered by Hep10@Gel after 2 hours. In addition, 21.3% of IL-1, 24.7% of TGF-β, and 30.7% of PDGF-BB were sequestered by Hep5@Gel. In contrast, less than 15% of the cytokines were bound to Hep0@Gel within the first 2 hours, and there was merely a slight further increase until 24 hours. After 24 hours of incubation, Hep10@Gel scavenged the most cytokines, with 65.3 ± 5.3% of IL-1, 45.7 ± 2.2% of TGF-β, and 57.6 ± 3.3% of PDGF-BB. The strong interaction between cytokines and Hep@Gel with a higher content of heparin was in line with previous studies, where IL-1, TGF-β, and PDGF-BB were reported to have the robust heparin-binding property (*37*).

Besides the cytokine-capturing ability of hydrogels, it is equally crucial that hydrogel should limit the diffusion of cytokines to sufficiently block the influx of cytokines. Compared with Hep0@Gel, Hep10@Gel displayed the superior capability to restrain the diffusion of IL-1, TGF-β, and PDGF-BB in the hydrogel (Fig. 4, B to D), with the bound cytokines mainly confined to the hydrogel surface (~50 μm), suggesting the enhanced capturing and reduced diffusion of cytokines in Hep10@Gel.

To simulate the normal tear microenvironment of the ocular surface when cytokines fade away, Hep10@Gel with bound cytokines was immersed in normal saline, and the long-term sequestration and degradation of cytokines in the hydrogels were illustrated. The hydrogel was then exposed to KCl to fully destroy the electrostatic interactions via the shielding effect (*38*), thus detecting the remaining captured cytokines (Fig. 4E). There was merely a slow and minimal spontaneous release of cytokines from Hep10@Gel in the saline, while more cytokines were still captured in the hydrogels (Fig. 4, F to H). After 1 day, 7.0 ± 4.4 pg of IL-1 was spontaneously released, with 25 times the amount of IL-1 remaining sequestered. Meanwhile, 54.4 ± 3.1 pg of TGF-β was released, while 67.4 ± 17.5 pg of TGF-β was still captured. When compared to the released PDGF-BB, 42 times the amount of PDGF-BB remained in the hydrogel. These results suggested the potent electrostatic sequestration of cytokines in Hep10@Gel, especially for IL-1 and PDGF-BB. As time went on, the total amount of cytokines (the sum of released and remaining cytokines) gradually decreased, revealing the progressive cytokine degradation. In short, our EBM-inspired design of bioactive Hep10@Gel was superior for scavenging inflammatory and fibrotic cytokines (Fig. 4I), thereby showing the potential to coordinate corneal stromal homeostasis upon injury.

### Inhibition of apoptosis and myofibroblast transition of keratocytes by Hep@Gel

**Fig. 5.**
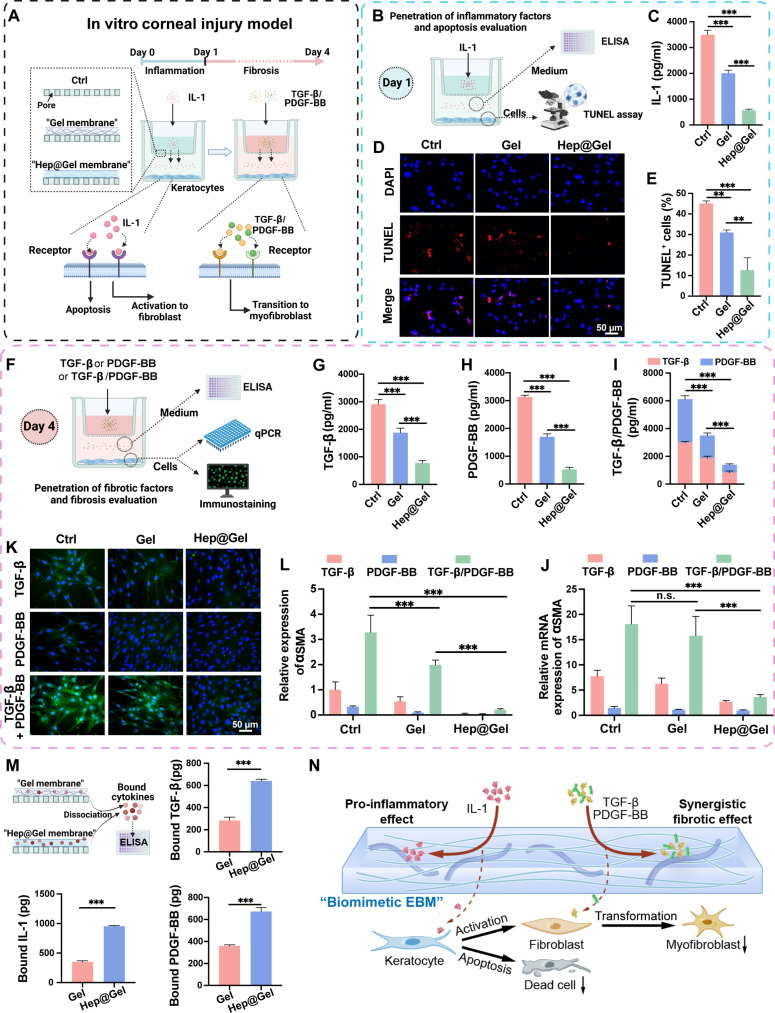
The barrier function of Hep@Gel in the in vitro model of corneal injury. (**A**) Scheme of the transwell system setup for the simulation of corneal epithelial-stromal injury to test the barrier function of Hep@Gel. Gel referred to Hep0@Gel, and Hep@Gel referred to Hep10@Gel. (**B**) Scheme of the detection of the amount of penetrated IL-1 in the lower chamber and apoptosis of keratocytes on day 1. (**C**) The concentration of IL-1 in the lower chamber (*n* = 3, one-way ANOVA test). (**D**) The terminal deoxynucleotidyl transferase–mediated deoxyuridine triphosphate nick end labeling (TUNEL) staining and (**E**) quantification of apoptotic keratocytes (*n* = 3, one-way ANOVA test). DAPI, 4′,6-diamidino-2-phenylindole. (**F**) Scheme of the detection of the amount of penetrated fibrotic cytokines in the lower chamber and myofibroblast transition of keratocytes on day 4. (**G** to **I**) The concentration of TGF-β, PDGF-BB, or TGF-β/PDGF-BB in the lower chamber [*n* = 3, one-way ANOVA test in (G) and (H); two-way ANOVA test in (I)]. (**J**) The mRNA expression of αSMA in keratocytes (*n* = 3, two-way ANOVA test). (**K**) Immunostaining and (**L**) quantification of αSMA in keratocytes (*n* = 3, two-way ANOVA test). (**M**) Quantification of bound cytokines in the hydrogel membrane. KCl was used to destroy the electrostatic interactions (*n* = 3, Student’s *t* test). (**N**) Scheme of the underlying mechanism of Hep@Gel to inhibit apoptosis and myofibroblast transition of keratocytes. **P < 0.01 and ***P < 0.001. n.s., not significant.

The EBM works as a barrier to limit the penetration of cytokines from the epithelium into the stroma (*27*). Encouraged by the robust cytokine-capturing ability of Hep@Gel, the barrier function of the bioactive hydrogel was evaluated in the in vitro corneal injury model, where exogenous inflammatory and fibrotic cytokines were sequentially used to simulate the cytokines released from the epithelium to trigger the wound healing cascade. The polycarbonate membrane of the transwell insert with pores was considered as the defective EBM, while hydrogels were used to restore the barrier (Fig. 5A). Accordingly, the inflammation-induced apoptosis of keratocytes was detected at the early stage, and the fibrotic cytokine-induced myofibroblast transition of keratocytes was evaluated later. Afterward, Gel referred to Hep0@Gel, and Hep@Gel referred to Hep10@Gel for convenience.

To simulate the early stage of corneal injury, the penetration of inflammatory cytokines into the lower chamber and the corresponding apoptosis of keratocytes were determined (Fig. 5B). Compared with the Ctrl group, Gel reduced IL-1 penetration by 42.8%, while Hep@Gel blocked 83.8% of IL-1 into the lower chamber (Fig. 5C). Moreover, according to the terminal deoxynucleotidyl transferase–mediated deoxyuridine triphosphate nick end labeling (TUNEL) staining, keratocyte apoptosis was inhibited to a portion of 12.6 ± 6.0% in the Hep@Gel group, significantly lower than the portion in the Gel group (31.0 ± 1.2%) and the Ctrl group (45.1 ± 1.2%) (Fig. 5, D and E, and fig. S8).

To simulate the later stage of corneal injury and investigate the synergistic pro-fibrotic effect of TGF-β/PDGF-BB, the penetration of fibrotic cytokines into the lower chamber and the phenotype transition from keratocytes to myofibroblasts were examined (Fig. 5F). Similarly, Hep@Gel limited the penetration of most fibrotic cytokines (Fig. 5, G to I). The quantitative polymerase chain reaction (qPCR) results indicated the synergistic effect of TGF-β and PDGF-BB to promote myofibroblast phenotype, with a synergy index of 2.4 calculated from the α-smooth muscle actin (αSMA) expression of the Ctrl group (Fig. 5J). On the basis of the synergistic fibrotic effect of TGF-β/PDGF-BB, Hep@Gel with its robust cytokine-capturing ability was supposed to efficiently inhibit the myofibroblast transition of keratocytes. Of note, under the stimulation of TGF-β/PDGF-BB, Hep@Gel showed the lowest relative mRNA expression of αSMA (3.6 ± 0.5), compared to the Gel (15.8 ± 3.8) and Ctrl (18.1 ± 3.6) groups. The immunostaining results of αSMA were consistent with the qPCR results (Fig. 5, K and L, and fig. S9). Moreover, keratocytes of the Hep@Gel group showed the highest mRNA expression of keratocyte-specific marker KERATOCAN (fig. S10), confirming the suppressed phenotype transition from keratocytes to myofibroblasts due to the potent barrier function of Hep@Gel.

Besides, the amounts of bound cytokines in the hydrogel membrane were measured (Fig. 5M). Without a doubt, Hep@Gel sequestered more cytokines than Gel, with 2.7 times higher amounts of IL-1, 2.3 times higher amounts of TGF-β, and 1.9 times higher amounts of PDGF-BB. Overall, the assumed barrier function of bioactive Hep@Gel was proved in the in vitro model of corneal injury, which effectively restored the impaired function of the EBM and down-regulated the wound healing cascade via restricting the penetration of cytokines through the hydrogel, laying a solid foundation for in vivo corneal repair (Fig. 5N).

### Wound healing promotion and vision quality improvement by Hep@Gel in rabbit corneal defects

Before in vivo application, the biocompatibility of Hep@Gel was examined in detail. Both HCECs and keratocytes seeded on the Gel and Hep@Gel exhibited high viability with a value over 95% (figs. S11 and S12). In addition, the hydrogel extraction showed almost no cytotoxicity for HCECs, keratocytes, lens epithelial cells, and retinal pigment epithelial cells (figs. S13 to S17). Moreover, the in vivo application of Hep@Gel and Gel on the ocular surface turned out to be biocompatible according to hematology examinations and histological sections of vital organs (figs. S18 and S19).

**Fig. 6.**
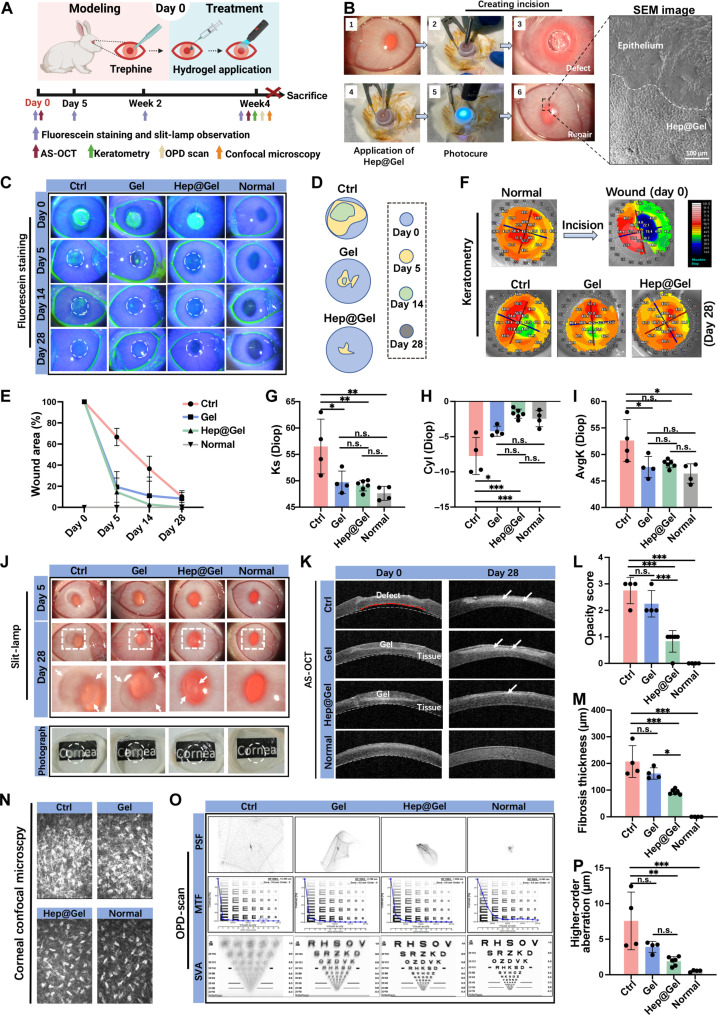
Wound healing promotion and scarring prevention by Hep@Gel in a rabbit corneal defect model. (**A**) The time axis to evaluate the therapeutic effect of Hep@Gel. (**B**) In vivo application of Hep@Gel adhesives. (**C**) Evaluation of corneal wound healing by fluorescein staining. The green area indicated the epithelial defects. (**D**) Changes in wound area. (**E**) Quantification of corneal wound healing. (**F**) Corneal keratometry and (**G** to **I**) quantification of the anterior corneal curvature. (**J**) Evaluation and (**L**) quantification of corneal opacity by slit lamp examination. A higher opacity score suggested more severe opacity, which was scored on day 28 after wounding. (**K**) Anterior segment optical coherence tomography (AS-OCT) of corneas. White dashed lines depicted the normal posterior corneal curvature, while the red dashed line showed the abnormal curvature. White arrows indicated the fibrosis tissues. (**M**) Quantification of corneal fibrosis thickness on day 28 after wounding according to the AS-OCT results. (**N**) Confocal microscopy of corneal stromal layer on day 28 after wounding. Hyperreflective dense deposits indicated stromal fibrosis. (**O**) OPD-scan results on day 28 after wounding. (**P**) The higher-order aberration of corneas according to the OPD-scan results. *n* = 4 for the Ctrl, Gel, and Normal groups, and *n* = 6 for the Hep@Gel group. One-way ANOVA test, *P < 0.05, **P < 0.01, and ***P < 0.001.

As a biocompatible hydrogel, Hep@Gel was further evaluated for its therapeutic effect on corneal injury (Fig. 6A). Upon creation of corneal incisions, the liquid Hep@Gel precursor was applied to the defect and then photocured. There was no visible boundary between the hydrogel and the adjacent tissue, facilitating the proper biointegration (Fig. 6B). Of note, the repaired cornea maintained transparency because of the transparency of Hep@Gel.

The corneal wound healing rate was analyzed in the following days. Both Gel and Hep@Gel accelerated the epithelial healing, and there remained only ~20% size of defects in these two groups on day 5 after wounding (Fig. 6, C to E). In contrast, the epithelial healing was slow in the Ctrl group with about 40% of the defect area remaining even on day 14 after wounding. The anterior corneal curvature became irregular upon corneal injury, which was visibly improved on day 28 after wounding especially in the Hep@Gel group (Fig. 6F). Compared with that of the Ctrl group, the anterior corneal curvature of the Gel group and the Hep@Gel group was more analogous to that of normal corneas (Fig. 6, G to I). Because both Gel and Hep@Gel provided collagen-derived gelatin to fill the defects and support the epithelial healing, it seemed reasonable that the corneal curvature was more regular in these two groups.

Previous studies usually overlooked corneal scarring, and, herein, the corneal fibrosis was extensively evaluated. The slit-lamp results demonstrated progressive corneal fibrosis in the Ctrl group from day 5 to day 28 after wounding (Fig. 6J). Gel slightly reduced fibrosis, while Hep@Gel prominently inhibited the fibrosis, achieving the lowest opacity score and the smallest haze area among the groups of wounded corneas (Fig. 6L and fig. S20). Besides, the anterior segment optical coherence tomography (AS-OCT) results revealed that both hydrogels improved the mechanical strength of the wounded cornea to withstand intraocular pressure and restore the abnormal posterior corneal curvature on day 0 (Fig. 6K), consistent with the findings in Fig. 3H that Hep@Gel had mechanical properties similar to those of the native cornea. On day 28 after wounding, minor hyperreflective fibrosis deposits were identified in AS-OCT of the Hep@Gel group, with the minimum fibrosis thickness (93.0 ± 9.3 μm) compared to that of Ctrl (207.3 ± 59.6 μm) and Gel (162.3 ± 21.3 μm) (Fig. 6M). The confocal microscopy further confirmed fewer stromal fibrotic deposits in the Hep@Gel group (Fig. 6N), with no obvious morphology difference of epithelial and endothelial layers among all groups (fig. S21). These results implicated that fibrosis mainly involved the stromal layer and Hep@Gel could effectively prevent the formation of excessive fibrotic tissues after corneal injury. Visual quality was another major concern related to corneal injury. Hep@Gel-treated corneas exhibited better simulated visual acuity (SVA) (Fig. 6O), and their higher-order aberration (1.9 ± 0.6 μm) was close to that of normal corneas (0.5 ± 0.1 μm), different from that of the Ctrl group (7.6 ± 4.1 μm) and the Gel group (3.9 ± 0.8 μm) (Fig. 6P and fig. S22).

Collectively, although both hydrogels facilitated corneal wound healing, Hep@Gel could better inhibit fibrosis and improve visual quality, implying the vital role of the barrier function of Hep@Gel. The underlying mechanism was further explored later.

### Wound healing cascade down-regulated by Hep@Gel to coordinate stromal homeostasis

**Fig. 7.**
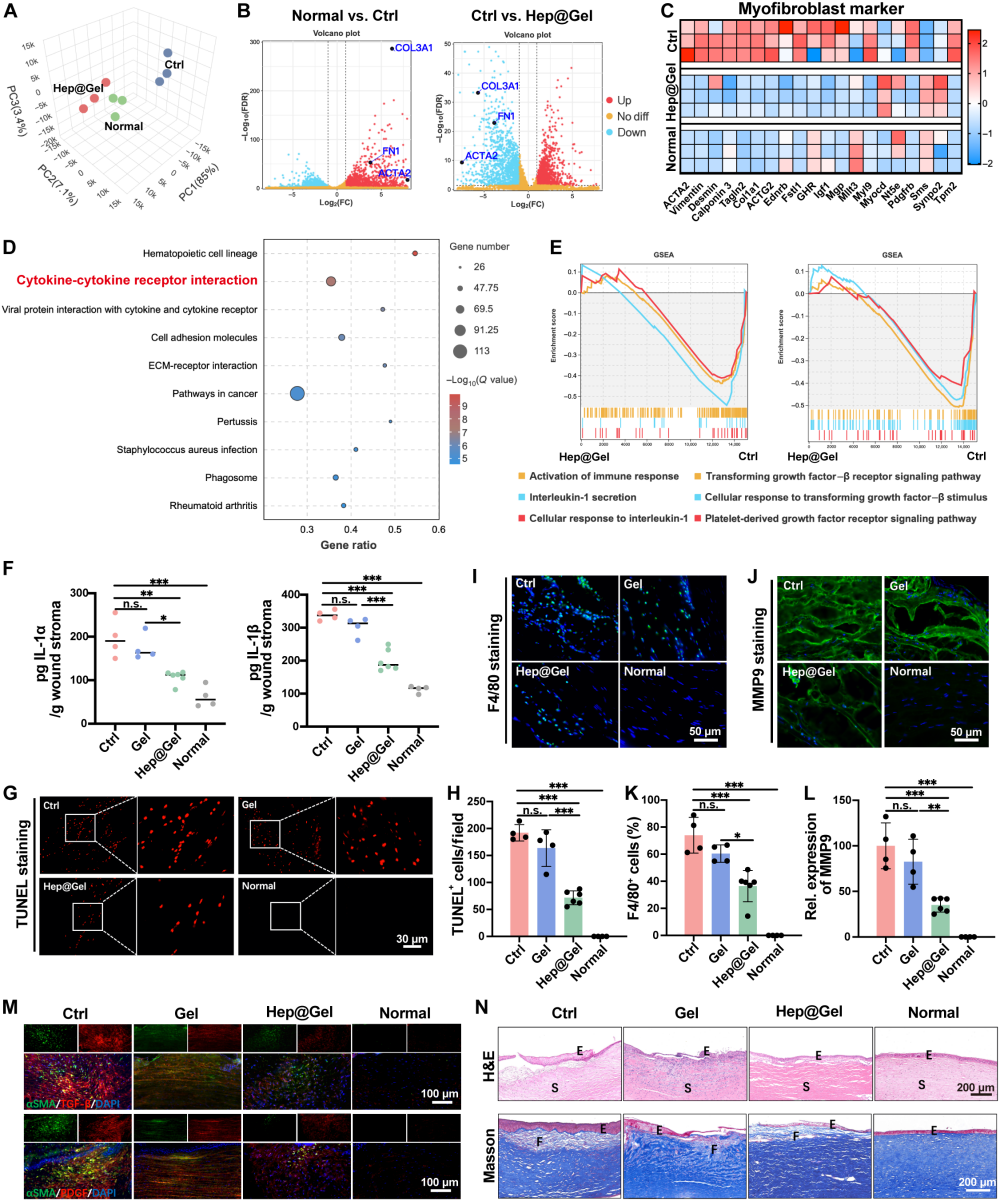
Corneal scarring inhibition by Hep@Gel via blocking the influx of inflammatory and fibrotic cytokines into the stroma. (**A**) Principal components analysis (PCA) plot of RNA-seq data. PC, principal component. (**B**) The volcano plot of RNA-seq data with fibrosis-related genes labeled. FC, fold change; FDR, false discovery rate. (**C**) Heatmap of genes related to fibrosis. (**D**) The top 10 enriched Kyoto Encyclopedia of Genes and Genomes pathways between the Ctrl group and the Hep@Gel group. (**E**) Gene set enrichment analysis (GSEA) analysis revealed the inhibition of inflammation-related pathways and fibrosis-related pathways in the Hep@Gel group. (**F**) Concentrations of IL-1α and IL-1β in the corneal tissue on day 3 after wounding. (**G**) Detection and (**H**) quantification of apoptosis in corneal stroma on day 3 after wounding. (**I**) Detection and (**K**) quantification of F4/80 expression in corneal stroma on day 3 after wounding. (**J**) Detection and (**L**) quantification of matrix metalloproteinase 9 (MMP9) expression in corneal stroma on day 3 after wounding. (**M**) Immunostaining of fibrotic cytokines and fibrosis tissue in the corneal stroma on day 28 after wounding. (**N**) Representative images of hematoxylin and eosin (H&E) staining and Masson staining on day 28 after wounding. E referred to the epithelium, S referred to the stroma, and F referred to the fibrosis tissues. *n* = 4 for the Ctrl, Gel, and Normal groups, and *n* = 6 for the Hep@Gel group. One-way ANOVA test, *P < 0.05, **P < 0.01, and ***P < 0.001.

RNA sequencing (RNA-seq) was used to explore the underlying mechanism involved in the scarless repair of Hep@Gel-treated corneas. The principal components analysis plot showed a similar gene expression pattern between Hep@Gel-treated corneas and normal corneas, which was different from that of untreated corneas (Fig. 7A). Specifically, the up-regulated fibrosis genes including actin alpha 2 (ACTA2), fibronectin 1 (FN1), and collagen type III alpha 1 (COL3A1) in the Ctrl group were suppressed in Hep@Gel-treated corneas (Fig. 7B). Moreover, compared with the Ctrl group, decreased myofibroblast phenotype was found in the Hep@Gel group (Fig. 7C), suggesting the inhibited myofibroblast phenotype transition in Hep@Gel-treated corneas. The heatmap results also showed delayed corneal epithelial maturation in the Ctrl group due to the low healing rate (fig. S23).

Next, altered singling pathways were investigated between the Ctrl group and the Hep@Gel group. The signaling pathway of cytokine-cytokine receptor interaction was enriched among the top 10 Kyoto Encyclopedia of Genes and Genomes pathways (Fig. 7D), and Gene Ontology analysis also revealed the altered cell surface receptor signaling pathway (fig. S24), suggesting the potential role of cytokine-cytokine receptor interaction in corneal repair. To figure out which cytokines determined the outcome of corneal repair, gene set enrichment analysis (GSEA) analysis was subsequently performed. Pathways related to secretion of chemokine IL-1, cellular response to IL-1, and activation of immune response were down-regulated in the Hep@Gel group. Besides, pathways of TGF-β receptor signaling, PDGF receptor signaling, and cellular response to TGF-β stimulus were also suppressed in Hep@Gel-treated corneas (Fig. 7E). Consequently, Hep@Gel was supposed to effectively block the influx of IL-1, TGF-β, and PDGF-BB, thus down-regulating the wound healing cascade.

To verify the above hypothesis, the influx of IL-1 at an early stage of corneal repair was examined. On day 3 after wounding, significantly decreased concentrations of IL-1α and IL-1β were detected in Hep@Gel-treated corneas (IL-1α, 114.1 ± 22.4 pg/g; and IL-1β, 187.2 ± 32.6 pg/g) compared to those of untreated corneas (IL-1α, 190.2 ± 44.9 pg/g; and IL-1β, 337.1 ± 15.7 pg/g) and Gel-treated corneas (IL-1α, 163.2 ± 30.1 pg/g; IL-1β, 312.8 ± 29.0 pg/g), indicating more effective sequestration of IL-1 in Hep@Gel (Fig. 7F). Accordingly, the smallest amounts of apoptotic cells were found in Hep@Gel-treated corneas (Fig. 7, G and H). The substantial influx of IL-1 was known to strongly trigger the immune response. Herein, Hep@Gel could vigorously reduce the infiltration of F4/80^+^ macrophages (Fig. 7, I and K) and attenuate the overexpression of matrix metalloproteinase 9 (MMP9) (Fig. 7, J and L) to inhibit the breakdown of the corneal stroma and the recruitment of more immune cells, which further confirmed the impeded influx of IL-1into the stroma by Hep@Gel.

Moreover, the influx of TGF-β /PDGF-BB at a later stage was also investigated. On day 28 after wounding, the colocalization of fibrotic cytokines and fibrotic tissues suggested that stromal fibrosis was driven by fibrotic cytokines. Notably, fewer TGF-β/PDGF-BB were detected in Hep@Gel-treated corneas, indicating the blocked influx of TGF-β/PDGF-BB by Hep@Gel (Fig. 7M). Accordingly, there were fewer αSMA^+^ and collagen type III (COL III)^+^ fibrotic tissues in the Hep@Gel group (figs. S25 and S26). In addition, both hydrogels (Gel and Hep@Gel) suppressed the epithelial hyperplasia compared to the Ctrl group (Fig. 7N), which might be attributed to the fact that hydrogels could promptly fill the corneal defect without the hyperplastic epithelium. In terms of stromal fibrosis, although Gel-treated corneas showed fewer fibrotic tissues than the untreated corneas, only Hep@Gel-treated corneas confined the fibrotic tissues to the superficial stroma (Fig. 7N), validating the barrier function of Hep@Gel in vivo. In summary, bioactive Hep@Gel efficiently blocked the substantial influx of IL-1 and TGF-β/PDGF-BB throughout corneal repair, resulting in a depletion of the cytokines from wounded corneas, which considerably down-regulated the wound healing cascade and eventually promoted organized corneal repair.

### Therapeutic effect of Hep@Gel on corneal defects in nonhuman primates

**Fig. 8.**
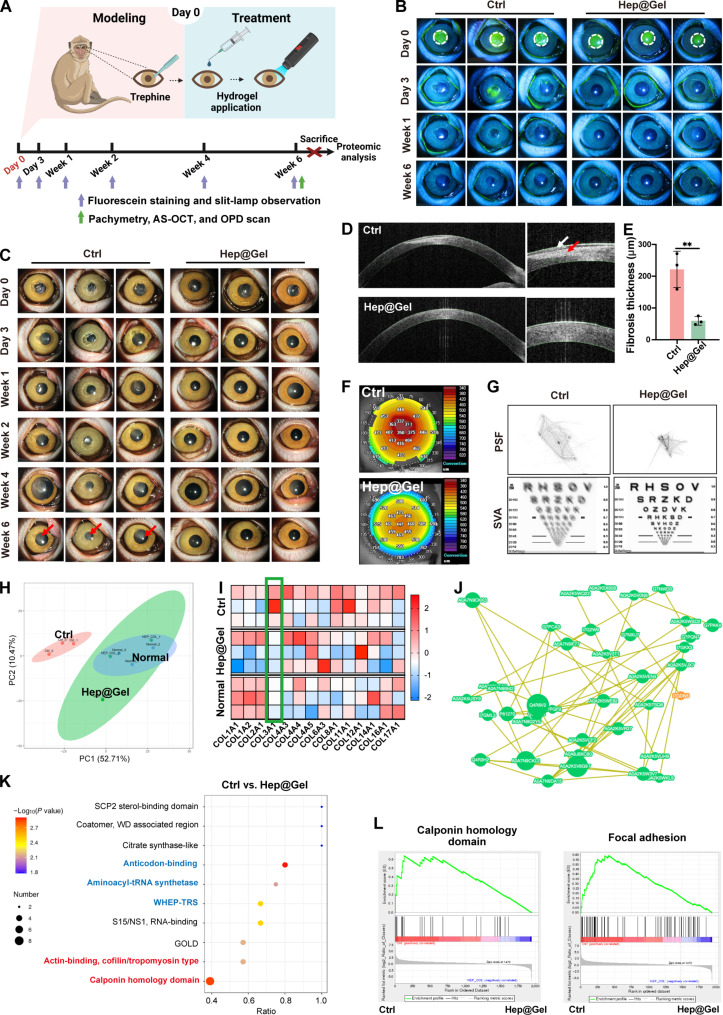
Therapeutic effects of Hep@Gel on the scarless corneal repair of cynomolgus monkeys. (**A**) The time axis to evaluate the therapeutic effect of Hep@Gel. (**B**) Evaluation of corneal wound healing. (**C**) Evaluation of corneal opacity. Red arrows indicated the corneal opacity. (**D**) AS-OCT images of corneas in week 6 after wounding. The white arrow pointed to the hyperplastic epithelium, and the red arrow showed the fibrosis tissue. (**E**) Quantification of corneal fibrosis thickness in week 6 after wounding (*n* = 3, Student’s *t* test, **P < 0.01). (**F**) Corneal pachymetry in week 6 after wounding. (**G**) OPD-scan of the corneas in week 6 after wounding. (**H**) PCA plot of proteomics data. (**I**) Heatmap of collagen proteins according to the proteomics data. (**J**) Protein-protein interaction networks and (**K**) protein domain enrichment analysis of differential proteins between the Ctrl group and the Hep@Gel group. (**L**) GSEA analysis revealed the inhibition of myofibroblast phenotype in the Hep@Gel group.

Seeking to bring our Hep@Gel closer to clinical relevance, the therapeutic efficacy of this hydrogel was further evaluated in cynomolgus monkeys with multiple examinations over a longer observation period compared to that of the rabbits (Fig. 8A). The corneal epithelial healing rate was determined during the follow-up. On day 3 after wounding, the epithelial healing was almost completed in Hep@Gel-treated corneas, and, in contrast, there remained defect areas in untreated corneas until day 7 (Fig. 8B and fig. S27). Besides, Hep@Gel-treated corneas maintained transparency, while the opacity of the untreated corneas worsened over time (Fig. 8C). In week 6 after wounding, the Hep@Gel group had a mean opacity score of 0.3, 10 times lower than that of the Ctrl group (fig. S28). Moreover, AS-OCT images showed the hyperplastic epithelium and the massive fibrotic deposits in the Ctrl group, and, instead, Hep@Gel markedly promoted scarless corneal repair with decreased fibrosis thickness (59.7 ± 13.9 μm), lower than that of the untreated corneas (221.7 ± 57.2 μm) (Fig. 8, D and E, and fig. S29). The corneal thickness measured by pachymetry in week 6 after wounding showed the restored thickness of the wounded corneas by Hep@Gel (Fig. 8F and figs. S30 and S31). Last, the wavefront aberration and visual quality of cynomolgus monkey corneas were measured in week 6 after wounding. The SVA in Hep@Gel-treated corneas was visibly improved with decreased wavefront aberrations compared to the Ctrl group (Fig. 8G figs. S32 and S33). Collectively, evaluations from multiple aspects suggested that Hep@Gel could effectively promote corneal wound healing, inhibit corneal scarring, and improve SVA in nonhuman primates.

Furthermore, corneas were collected for proteomics analysis in week 6 after wounding. Compared with untreated corneas, the protein expression profiling of Hep@Gel-treated corneas was more analogous to that of the normal corneas (Fig. 8H and fig. S34). Considering that the corneal stroma was mainly composed of collagens, the protein profiling of collagens was investigated. Specifically, the expression of COL3A1, the collagen closely relevant to fibrosis, was down-regulated in the Hep@Gel group compared to that in the Ctrl group (Fig. 8I), suggesting that the overproduction of fibrosis-related ECM was suppressed in Hep@Gel-treated corneas. The protein-protein interaction further revealed that numerous proteins involved in the function of ribosomal structure and biogenesis, translation, and protein turnover were generally inhibited in the Hep@Gel group (Fig. 8J and table S1), which might account for the fewer fibrosis deposits in Hep@Gel-treated corneas.

Given that myofibroblasts produce excessive ECM to heal the wound roughly, the phenotype transition from keratocytes to myofibroblasts should contribute to fibrosis deposits. Protein domains as markers for myofibroblasts, such as tropomyosin type and calponin homology domain, were enriched between the Hep@Gel group and the Ctrl group, implying that the myofibroblast phenotype in wounded corneas was greatly inhibited by Hep@Gel (Fig. 8K). Moreover, domains related to protein production, including anticodon-binding, aminoacyl-tRNA synthetase, and WHEP-TRS, were also enriched, indicating the attenuated production of fibrotic components in the Hep@Gel group, which was consistent with the GSEA plots (Fig. 8L). Thinking that the impaired EBM upon corneal injury leads to the influx of multiple cytokines to induce the myofibroblast phenotype, Hep@Gel was supposed to be a barrier to resist the impact of excessive cytokines.

In conclusion, Hep@Gel could facilitate scarless corneal repair of cynomolgus monkeys via the suppression of myofibroblast phenotype transition and fibrotic deposits, which was attributed to the barrier function of Hep@Gel to coordinate stromal homeostasis.

## DISCUSSION

By 2020, more than 230 million people worldwide suffer from visual impairment, and, among them, around 39 million people are blind (*39*). More than 12 million people belong to corneal blindness, accounting for one-third of blind people (*5*, *6*). Corneal blindness is closely related to scarring that forms due to the unrestrained wound healing cascade. However, the relatively high risk of steroid-induced complications and the severe shortage of available donor corneas make it tricky to treat corneal scarring. Although ECM-based hydrogels show therapeutic effects on corneal repair (*40*, *41*), the accompanied scarring is usually overlooked. Accordingly, we proposed the biomimetic EBM strategy biologically inspired by native EBM, which potently down-regulated the wound healing cascade without additional cells or drugs, working as an all-around solution for corneal scarring.

The scaffolding GelMA hydrogel was synthesized on the basis of gelatins, which are derived from collagens, the component of both the EBM and the corneal stroma. A notable feature of Hep@Gel is its adhesive property. Because of the disulfide bond–based adhesive mechanism (*42*), the hydrogel showed robust adhesion to tissues, thus preventing the detachment of hydrogels from the ocular surface despite the frequent blinking. Besides, the proper hydrogel-cornea integration contributed to the good biocompatibility of hydrogels in vivo, with no red inflamed eye observed in the rabbits and cynomolgus monkeys. Furthermore, our photocurable Hep@Gel enabled prompt corneal structural support via ECM replenishment and effectively established a barrier resembling the native EBM using negatively charged heparin with substantial sulfate anion. Because of the robust cytokine-capturing ability of Hep@Gel and the limited cytokine diffusion, bioactive Hep@Gel was supposed to efficiently block the influx of inflammatory and fibrotic cytokines from the epithelium into the stroma.

As a proof of concept, the wound healing cascade was down-regulated by Hep@Gel. At the early stage of wound healing, fewer cytokines of IL-1α and IL-1β infiltrate the stroma due to the barrier function of Hep@Gel, resulting in less apoptosis of stromal cells. Besides, the decreased concentration of IL-1 contributed to fewer recruited macrophages from the limbal vessel, providing a relatively reparative immune microenvironment, which is known to promote wound healing (*43*, *44*). TGF-β is a master regulator featured with its potent pro-fibrotic effects in a variety of tissues, including the cornea (*45*, *46*). However, according to our findings about Hep@Gel, the binding ability of TGF-β was inferior to that of PDGF-BB. Regarding the prominently inhibited scarring in Hep@Gel treated corneas, it is noteworthy to highlight the synergistic effect of TGF-β and PDGF-BB confirmed in our study, with a calculated synergistic index of 2.4. Thereby, the reduced concentration of both TGF-β and PDGF-BB in the stroma markedly down-regulated the wound healing cascade in the later stage, synergizing to strongly suppress the corneal scarring.

The treatment of corneal scarring includes two aspects. Upon injury, the formation of scarring should be prevented. Once the scarring has formed, corneal debridement and subsequent lamellar keratoplasty need to be executed. Because our animal models simulate the conditions of both corneal injury and post-corneal debridement, Hep@Gel proved to be an all-around strategy for scarring treatment, which was effective for scarring prevention and substituting for lamellar keratoplasty to remove already existing scarring, thus greatly alleviating over-dependence on donor corneas.

Given that the EBM is an essential structure within multicellular organisms (*47*–*49*), the concept of the biomimetic EBM may extend beyond corneal injury. For instance, numerous studies have revealed that the regeneration of the functional epidermis relies on the reconstitution of the EBM (*50*), indicating the potential practice of the biomimetic EBM strategy. Moreover, our bioactive Hep@Gel could eliminate concerns about batch-to-batch variation and biosafety of stem cells and avoid the off-target effects of drugs.

One limitation of our study is that we have not conducted clinical trials yet. Of note, GelMA has already been recognized to be a safe category by the Food and Drug Administration (*51*), and heparin is widely used as a clinical anticoagulant drug, suggesting the safety of Hep@Gel. Moreover, considering the remarkable therapeutic effect of Hep@Gel confirmed by models of nonhuman primates, we hold a positive attitude toward possible clinical trials. Clinical translation of Hep@Gel could be facilitated by the safety of hydrogels. As a following step, it is critical to establish a good manufacturing practice–grade Hep@Gel as a prerequisite for clinical trials in humans, which will also be our future work. Besides, because the native EBM is a quite thin layer that efficiently binds cytokines to maintain homeostasis, further optimization of Hep@Gel could be performed to minimize cytokine diffusion as much as possible, which is exactly what we plan to do in the future.

## MATERIALS AND METHODS

### Experimental design

This study aims to develop corneal EBM-inspired bioactive Hep@Gel for scarless corneal repair. Human corneal EBM was investigated to inspire the design of bioactive hydrogels. The transparency and mechanical properties of Hep@Gel were studied using the microplate reader and rheometer, and the tissue adhesive performance was also determined. Then, the cytokine-capturing ability of Hep@Gel was examined to test whether it reduced the diffusion of IL-1, TGF-β, and PDGF-BB. The in vitro corneal injury model constructed from transwell systems was used to reveal the barrier function of Hep@Gel to inhibit apoptosis and myofibroblast transition of keratocytes. After being evaluated for biocompatibility both in vitro and in vivo, the hydrogel’s therapeutic effect was further extensively examined in rabbit and nonhuman primate models of corneal injury, and the underlying mechanism was illustrated.

### Investigation of the human corneal EBM

The remaining tissues after corneal transplantation were collected for analysis with informed consent from donors or their legally authorized representatives, approved by the Second Affiliated Hospital Ethics Committee, Zhejiang University, School of Medicine (2024-520). Some human corneal tissues were fixed with glutaraldehyde solution, and the microstructure of the corneal EBM was observed by TEM after the tissue section. Some corneal tissues were fixed by formalin, dehydrated, embedded in paraffin, and sectioned. PAS staining and immunostaining were performed to detect the components of the corneal EBM.

### Docking analysis of various cytokines to heparin

On the basis of previously published protein structures from the UniProt, the computational docking analysis of IL-1, TGF-β, and PDGF-BB to heparin was performed using MOE2019 software. The Protein Data Bank ID for IL-1, TGF-β, and PDGF-BB was 2KKI, 1KLA, and 1PDG, respectively. To ensure the rationality of the docking results, under the premise of low binding energy, conformations with most interactions between ligands and proteins were chosen for display.

### Synthesis of GelMA

GelMA was prepared from type A porcine skin gelatin. Briefly, gelatin was dissolved in PBS at a final concentration of 10% (w/v) at 60°C. Then, methacrylic anhydride was added dropwise to the solution at 50°C to achieve a 20% (v/v) methacrylic anhydride solution. After 1 hour of stirring, warm PBS (40°C) was used to dilute the reaction system (5×) to stop the reaction. The resultant mixture was dialyzed [molecular weight cutoff (MWCO), 10,000] against deionized water at 40°C before being lyophilized. Last, the lyophilized GelMA was kept at 4°C for storage. GelMA was analyzed with ^1^H nucleic magnetic resonance (^1^H NMR) measurement (DMX-500, BRULCER).

### Synthesis of HepSH

HepSH was prepared from heparin. Briefly, 1 g of heparin [molecular weight (MW), 14,000], 768 mg (4 mmol) of 1-(3-dimethylaminopropyl)-3-ethylcarbodiimide, and 612 mg (4 mmol) of 1-hydroxybenzotriazole hydrate were dissolved in deionized water, and the mixture was stirred for 2 hours. The reaction mixture was mixed with 900 mg (4 mmol) of cystamine dihydrochloride and stirred overnight. The mixture was dialyzed (MWCO, 3500) against deionized water for 1 day. Then, 1 g of dl-dithiothreitol was added to the mixture, and the solution was stirred for 1 day. The resultant mixture was dialyzed (MWCO, 3500) at pH 3 and lyophilized. Last, the lyophilized HepSH was stored at −20°C until further use. HepSH was analyzed with ^1^H NMR measurement and Ellman’s assay for thiolation determination.

### Preparation and characterization of Hep@Gel

LAP [0.25% (w/v)] was dissolved in deionized water as the photoinitiator solution. GelMA and HepSH were then dissolved in the photoinitiator solution, and the mixture was kept away from light. To obtain Hep@Gel hydrogels, the mixture was cross-linked using 365-nm ultraviolet light (XP104, AVENTK), with a power of 15 mW cm^−2^. The transparency of the hydrogels with varying ratios of GelMA and HepSH was determined according to measured light absorbance following the reported method (*52*). Briefly, 100 μl of hydrogel precursor solution was added to the 96-well plate and photocured. The absorbance of hydrogels was measured in the wavelength range of visible light (400 to 800 nm) using the microplate reader (M200 PRO NanoQuant, TECAN) with PBS as the blank. The transmittance was calculated using the following formula: *T* (%) = 1/10*^A^* × 100. *T* refers to the transmittance, and *A* refers to the absorbance. The rheological behavior of Hep@Gel was measured using a rheometer (HAAKE MARS 60, Thermo Fisher Scientific) based on a reported method (*52*). Hep@Gel precursor solution was photocured on a parallel plate. Frequency sweep tests were carried out from 0.1 to 10 Hz with a fixed strain of 1% at room temperature. The porous three-dimensional networks of Hep@Gel were observed with the SEM (SU8010, HITACHI) at 3.0 kV.

To determine the swelling ratios and EWC, 100 μl of hydrogel discs of Hep@Gel were incubated in PBS at room temperature. At predetermined times, the hydrogels were blotted with delicate task wipers and weighed as the swollen weight. The mass swelling ratio (w/w) was obtained using the following formula: swelling ratio (%) = (*W*_s_ − *W*_i_)/*W*_i_ × 100%. *W*_s_ refers to the swollen weight, while *W*_i_ refers to the initial weight before incubation in PBS. As for the EWC, EWC (%) = (*W*_e_ − *W*_d_)/*W*_e_ × 100%. *W*_e_ refers to the equilibrium-swollen weight, and *W*_d_ refers to the lyophilized weight.

The capability of Hep@Gel to reinforce the impaired mechanical strength of rabbit corneas was evaluated. Briefly, a 3-mm full-thickness incision was made in the center of the corneal button. The incision was either filled with 8 μl of Hep@Gel or left untreated. Corneas were put on the parallel plate, and frequency sweep tests were carried out from 0.1 to 10 Hz at 1% strain (HAAKE MARS 60, Thermo Fisher Scientific).

The radar chart with the index of transparency, mechanical strength, and water content was drawn to visually display the similarity between Hep10@Gel and the native cornea. The quantitative data for transparency and water content of the native cornea were from the previously reported studies (*20*, *53*).

### Tissue-adhesive study

The tissue-adhesive properties of Hep@Gel were evaluated as follows. First, fresh porcine skin from local butcher shops was washed several times, and subcutaneous fat was removed. Rhodamine B staining was added to the precursor solution to visually indicate the hydrogel. Precursor solution (200 μl) was added onto the surface of the porcine skin and was photocured without interference. Then, the interferences of fold and torsion were performed to examine the adhesive flexibility of hydrogels.

To further test the adhesive properties, lap-shear adhesion experiments were performed. Hep@Gel or fibrin glue (20 μl) was used to adhere two pieces of glass slides together with an overlap area of 1 cm by 2.5 cm. The shear strength was measured with the mechanical tester (Z020, Zwick/Roell), and the tensile stress was set at 1 mm/min. The shear strength was calculated from the maximum stress during the test process.

Further, 30 μl of precursor solution was injected into the overlap area (3 mm by 10 mm) between two pieces of the porcine cornea (half-cornea) and was photocured. Meanwhile, fibrin glue was used as a control. The adhesive strength was tested with the mechanical tester at a strain rate of 1 mm/min. The adhesive strength was also recorded at the maximum tensile stress.

### Ex vivo burst pressure tests

The ex vivo sealing ability of Hep@Gel hydrogels was determined with the burst pressure tests on porcine corneal tissues as shown in Fig. 3 (P and Q). Briefly, a 3-mm full-thickness punch injury was made in the cornea, and 30 μl of the hydrogels or fibrin glue was used to seal the corneal perforation. Subsequently, the corneas were mounted on the artificial anterior chamber, which was connected to a syringe pump (ISPLab01, DK Infusetek) and a digital pressure indicator (DPI705, Druck). Blue-colored normal saline (premixed with aniline blue) was injected into the artificial anterior chamber at 1 ml/min to increase the intraocular pressure. The maximum pressure was recorded as the burst pressure.

### Cytokine binding to Hep@Gel

A mixture of cytokines was prepared as follows to characterize the cytokine binding capability of Hep@Gel. IL-1, TGF-β, and PDGF-BB were added to the Dulbecco’s modified Eagle’s medium/F12 medium containing 0.1% (w/v) bovine serum albumin to reach a final concentration of 10 ng/ml for each cytokine. Hep@Gel (20 μl) was incubated in 1 ml of this mixture of cytokines at room temperature for 24 hours. At predetermined times, the remaining cytokines in the supernatants were collected for quantification by enzyme-linked immunosorbent assay (ELISA). A control group without hydrogel was used for normalizing the concentrations thinking of the cytokine degradation over the incubation period.

Further, the cytokine-bound Hep0@Gel and Hep10@Gel were sectioned for immunostaining. Briefly, the hydrogels were embedded in an optimal cutting temperature compound (Tissue-Tek, SAKURA) and were sliced into 7-μm transverse sections using a cryostat machine (NX50, Thermo Fisher Scientific). Then, the sections were permeabilized, blocked, and incubated with primary antibodies. On the following day, sections were subjected to secondary antibodies. Last, a fluorescence microscope was used to observe the immunostaining results.

### Assessment of the long-time sequestration and degradation of cytokines in Hep@Gel

Hep@Gel (20 μl) was incubated in 1 ml of the abovementioned mixture of IL-1, TGF-β, and PDGF-BB (10 ng/ml) at room temperature for 24 hours. The hydrogel was then immersed in 1 ml of normal saline to simulate the cytokine release from Hep@Gel under the normal tear microenvironment of the ocular surface. On days 1, 3, and 7, the cytokines released in the normal saline were analyzed by ELISA. Immediately after the collection of normal saline, hydrogels were transferred to 400 μl of KCl solution (500 mM) for 30 min to strongly destroy the electrostatic interactions between cytokines and hydrogels via the shielding effect (*54*). The cytokines released in the KCl solution were also analyzed by ELISA to detect the remaining bound cytokines in the hydrogels.

### Evaluation of the barrier function of Hep@Gel

A corneal injury model was established in vitro using a transwell system. The polycarbonate membrane (8-μm pore size) of a 24-well transwell insert was used to simulate the impaired corneal EBM. Gel hydrogels or Hep@Gel hydrogels (10 μl) were applied to the surface of the polycarbonate membrane to function as a barrier to rescue the impaired EBM.

Keratocytes were cultured in a well of 24-well plate with a density of 1 × 10^5^ cells/ml in 1 ml of commercial medium. Then, 300 μl of medium containing IL-1 (20 ng/ml) was added to the transwell insert, simulating the inflammatory cytokines released from the injured epithelium. After incubation for 1 day, the culture medium of the lower chamber was collected to evaluate the penetration of IL-1 through the membrane using the ELISA kits. Besides, the apoptosis of keratocytes was assessed by TUNEL assay. Then, the medium of the transwell insert was changed to 300 μl of medium containing TGF-β, PDGF-BB, or TGF-β/PDGF-BB (20 ng/ml), simulating the fibrotic cytokines released from the injured epithelium. After incubation for another 3 days, the culture medium of the lower chamber was collected to evaluate the penetration of fibrotic cytokines through the membrane using the ELISA kits. Besides, keratocytes were analyzed for the expression of fibrosis markers by qPCR and immunostaining.

To investigate the synergistic pro-fibrotic effect of TGF-β and PDGF-BB, the synergy index was calculated according to the qPCR results of αSMA expression in keratocytes exposed to TGF-β/PDGF-BB, TGF-β, and PDGF-BB, respectively. The formula was as follows (*55*): synergy index = (*A* − 1)/[(*B* − 1) + (*C* − 1)]. *A*, *B*, and *C* referred to the pro-fibrotic effect of TGF-β + PDGF-BB, TGF-β, and PDGF-BB, respectively. If the synergy index > 1, then it indicates the synergetic effects of TGF-β and PDGF-BB.

The amount of bound IL-1, TGF-β, and PDGF-BB in the hydrogel membrane was also evaluated. Briefly, the transwell inserts were immersed in 500 mM KCl to strongly destroy the electrostatic interactions and promote the release of bound cytokines from the hydrogel membrane (*38*). The amount of bound IL-1, TGF-β, and PDGF-BB was further detected by ELISA.

### Rabbit corneal wound healing studies

The 2.5-kg male New Zealand rabbits were used for experiments, approved by the Second Affiliated Hospital Ethics Committee, Zhejiang University, School of Medicine (2022-157). The corneal trephine with a 3-mm diameter was used to conduct the lamellar keratectomy (a depth of ~30%), as previously reported (*22*). The rabbits with corneal injuries were then randomly divided into three groups. The Ctrl group: left untreated. The Gel group: The corneas were filled with 3 μl of Gel hydrogel. The Hep@Gel group: The corneas were filled with 3 μl of Hep@Gel hydrogel. Besides, the Normal group was set up: The corneas are normal without lamellar keratectomy. Levofloxacin eye drops were applied to all wounded eyes three times per day to prevent infection for 2 weeks.

A series of examinations were performed on the basis of our previously established studies (*22*). Corneas were stained by fluorescein sodium to evaluate the epithelial wound healing. Slit-lamp examinations (YZ5T, 66 VISION Technology) were used to observe and record the corneal opacity. AS-OCT (CASIA, TOMEY) and corneal confocal microscopy (HRT3, Heidelberg Engineering) were used to observe the hyperreflective deposits in the corneal stroma that are closely related to fibrosis. Keratometry examinations (CASIA, TOMEY) were performed to analyze the anterior corneal curvature to assess the degree of regularity of the regenerated cornea. OPD-scan (OPD-Scan III, NIDEK) was performed to detect the wavefront aberration to determine the visual quality.

On day 3, some rabbits were euthanized. To detect the amount of IL-1 in the wound area of the corneas, the tissue lysis of the wound area was prepared for ELISA quantification. The MMP9 antibody and the F4/80 antibody were used for immunostaining to assess the inflammatory responses. The TUNEL assay was used to detect the apoptosis in the corneal stroma. On day 28, all rabbits were euthanized for RNA-seq and histological examinations of the corneas.

### Cynomolgus monkey corneal wound healing studies

The male cynomolgus monkeys (3 to 3.5 years, weighing 3.5 ± 0.5 kg) were housed in individual cages and were used for experiments, approved by the Second Affiliated Hospital Ethics Committee, Zhejiang University, School of Medicine (2022-143). Monkeys were anesthetized by intramuscular Zoletil (5 mg kg^−1^ body weight) injection, and the lamellar keratectomy was conducted (~30% depth). The monkeys with corneal injuries were then randomly divided into two groups. The Ctrl group: left untreated. The Hep@Gel group: The corneas were filled with 3 μl of Hep@Gel hydrogel. Besides, the Normal group was set up: The corneas are normal without lamellar keratectomy. Levofloxacin eye drops were applied to all wounded eyes three times per day to prevent infection for 2 weeks. Fluorescein staining, slit-lamp observations, pachymetry, AS-OCT, and OPD-scan were performed to determine the therapeutic effect. After 6 weeks after wounding, all cynomolgus monkeys were euthanized for label-free proteomics of the corneas.

### Statistical analysis

Data were displayed as means ± SD. All experiments were independently performed at least three times, if not specifically indicated. Student’s *t* test, one-way analysis of variance (ANOVA), and two-way ANOVA were used to evaluate the differences between different groups. *P* < 0.05 was statistically significant. *P < 0.05; **P < 0.01; ***P < 0.001; n.s., not significant.
