# Supplementary material for: Biologically inspired bioactive hydrogels for scarless corneal repair
Source: Sci Adv. 2024 Dec 18;10(51):eadt1643. doi: 10.1126/sciadv.adt1643 (PMC11654680; doi:10.1126/sciadv.adt1643)
Supplement: Supplementary file 1 — Supplementary Methods Figs. S1 to S34 Tables S1 to S3 Legend for movie S1 [file sciadv.adt1643_sm.pdf]

Science Advances
AAAS

Supplementary Materials for

Biologically inspired bioactive hydrogels for scarless corneal repair

Jianan Huang *et al.*

Corresponding author: Ke Yao, xlren@zju.edu.cn; Haijie Han, hanhj90@zju.edu.cn

Sci. Adv. **10**, eadt1643 (2024)
DOI: 10.1126/sciadv.adt1643

The PDF file includes:

Supplementary Methods
Figs. S1 to S34
Tables S1 to S3
Legend for movie S1

Other Supplementary Material for this manuscript includes the following:

Movie S1

Supplementary Methods

Materials: Type A porcine skin gelatin, methacrylic anhydride, EDC, cystamine dihydrochloride, and Triton X-100 were purchased from Sigma-Aldrich. Heparin was bought from Millipore. HOBt and aniline blue were obtained from Aladdin Biochemical Technology. PBS, DTT, and KCl were purchased from Sangon Biotechnology. The PAS staining kit, TUNEL kit, and H&E staining kit were all from Beyotime Biotechnology. LAP was purchased from Yongqinquan Intelligent Equipment Co., Ltd. Rhodamine B staining, goat serum, DAPI dye, Calcein-AM/PI kit, and Masson staining kit were bought from Solarbio Science & Technology Co., Ltd. DMEM/F12 medium, fetal bovine serum, and penicillin-streptomycin were purchased from Thermo Fisher Scientific. IL-1, TGF-β, and PDGF-BB were purchased from Peprotech. Bovine serum albumin was from Fude Biological Technology. ELISA kits for IL-1, TGF-β, and PDGF-BB were purchased from Cusabio Technology. Levofloxacin eye drops were obtained from Santen Pharmaceutical. The culture medium for keratocytes was purchased from ScienCell Research Laboratories. E.Z.N.A.® Total RNA Kit I was from Omega Biotek. PrimeScript™ RT Master Mix and Takara TB Green™ Premix Ex Taq™ II were bought from Takara. CCK-8 kit was purchased from Yeasen. The primary antibodies used are listed in Table S2. The primers used are listed in Table S3.

Cell culture: HCECs, the human lens epithelium-derived cell line (SRA) and the retinal pigment epithelial cells (RPE) were all purchased from ATCC. HCECs were cultured in DMEM/F12 medium supplemented with 10% (v/v) fetal bovine serum and

1% (v/v) penicillin-streptomycin. SRA cells were cultured in DMEM (high glucose) supplemented with 20% (v/v) fetal bovine serum and 1% (v/v) penicillin-streptomycin. RPE cells were cultured in DMEM/F12 medium supplemented with 10% (v/v) fetal bovine serum and 1% (v/v) penicillin-streptomycin. Keratocytes were purchased from ScienCell Research Laboratories, and cells were cultured in the commercial culture medium. Cell culture dishes were placed in a 37 °C incubator (Midi 40, Thermo Fisher Scientific) with 5% CO_2, and the culture medium was changed every two days.

Wound scratch assay: HCECs were seeded in a 12-well plate at a density of 3×10^5 cells per well in 1 mL of culture medium, and the medium was changed every two days. Two crossed scratches were created in a confluent cell layer using a 200-μL pipette tip, and fresh medium was added to the well plate after the scratch. After 24 hours, the cell culture supernatants of HCECs with or without scratches were freshly collected to detect the secreted cytokines including IL-1, TGF-β, and PDGF-BB using ELISA kits according to the manufacturer's instructions.

Immunocytochemistry: For myofibroblast phenotype identification, keratocytes growing on glass slides were washed 3 times with PBS and fixed with 4% (w/v) paraformaldehyde for 15 minutes. Then, cell permeabilization was performed with 0.25% (v/v) Triton X-100 for 5 minutes, and the cells were blocked with 5% (v/v) goat serum and 1% (w/v) bovine serum albumin for 1 hour, followed by incubation with the primary antibody (αSMA) at a 1:200 dilution at 4 °C overnight. The next day, the cells were incubated with secondary antibodies for 1 hour. DAPI dye was used to

stain the nuclei. The cells were examined under an optical microscope (ECLIPSE-Ni, Nikon), and pictures were taken with NIS-Elements D 4.20.00 software.

Quantitative PCR (qPCR): Total RNA was extracted from cells with E.Z.N.A.® Total RNA Kit I. The quality of RNA samples was determined according to A260/A280 (values between 1.8 and 2.1). For each sample, 1 μg of RNA was used to synthesize cDNA using PrimeScript™ RT Master Mix. Then, quantitative PCR was performed with 40 amplification cycles with specific primers and Takara TB Green™ Premix Ex Taq™ II in a Real-Time PCR Detection System (CFX96, Bio-Rad). CT values were measured, and the expression of GAPDH was used as the control.

***In vitro* cytocompatibility assay**: Hydrogel was formed on a glass slide and then placed inside a 12-well plate. Then HCECs or keratocytes were added to the 12-well plate to cover the gel at a density of 1×10^5 cells per well. After 2 days, the viability of cells was determined with the live/dead assay using the Calcein-AM/PI kit according to the manufacturer's instructions. Cells growing on uncoated glass slides were used as controls (the Mock group). Besides, the cytocompatibility of the hydrogel extraction was also evaluated. Briefly, 20 μL of hydrogel was immersed in 1 mL of culture medium for 24 hours. The hydrogel extraction was used to culture HCECs, keratocytes, SRA, and RPE for 2 days. Then the viability of cells was determined by the live/dead assay and the CCK-8 assay according to the manufacturer's instructions. Cells cultured in the normal medium were used as controls (the Mock group).

***In vivo* biocompatibility assay**: The *in vivo* biocompatibility of hydrogels was determined using the corneal defect model of rabbits. The corneal trephine with a

3-mm diameter was employed to conduct the lamellar keratectomy (a depth of ~30%). The rabbits with corneal injuries were then randomly divided into 3 groups. The Ctrl group: left untreated. The Gel group: the corneas were filled with 3 μL of Gel hydrogel. The Hep@Gel group: the corneas were filled with 3 μL of Hep@Gel hydrogel. Besides, the Normal group was set up: the corneas are normal without lamellar keratectomy. On day 3 post-wounding, blood samples of various groups were collected for hematology examinations. The test index included the WBC, RBC, PLT, HGB and so on. On day 28 post-wounding, all rabbits were sacrificed and the vital organs (heart, liver, spleen, lung, and kidney) of various groups were collected for histological analysis.

Histological analysis: For H&E and Masson staining, the corneal tissues were fixed with 4% (w/v) paraformaldehyde overnight, embedded in paraffin, sliced into 4-μm thick sections, and stained with H&E and Masson's trichrome stains according to the manufacturer's instructions. For immunostaining, tissue sections were permeabilized, blocked, and incubated with primary antibodies. The next day, the sections were incubated with secondary antibodies, and the nuclei were stained with DAPI. Histological examination was performed under an optical microscope, and pictures were taken with NIS-Elements D 4.20.00 software.

The scoring system for clinical assessment of corneal opacity: 0: complete transparent; 1: slight opacity; 2: moderate opacity, but iris texture visible; 3: severe opacity, but pupil visible; 4: complete opacity and pupil not visible.

Supplementary figures

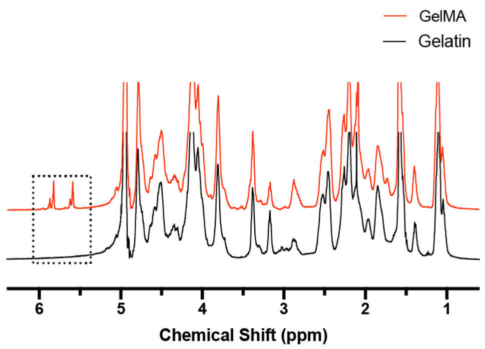

Fig. S1. ^{1}H-NMR spectra of gelatin and GelMA.

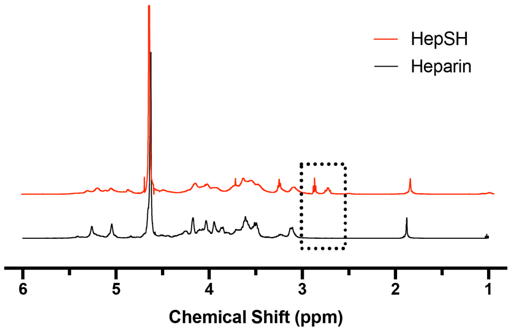

Fig. S2. ^{1}H-NMR spectra of heparin and HepSH.

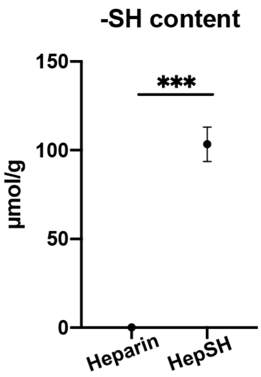

Fig. S3. Quantification of free thiols in heparin and HepSH by Ellman's assay (n = 3, Student's t-test, *** $P < 0.001$).

Gel time

GelMA (mg/mL)	HepSH (mg/mL)			
	0	5	10	20
0		liquid	liquid	liquid
100	10 ~ 15 s	10 ~ 15 s	10 ~ 15 s	semi-solid
200	5 ~ 10 s	5 ~ 10 s	5 ~ 10 s	5 ~ 10 s
300	0 ~ 5 s	0 ~ 5 s	0 ~ 5 s	0 ~ 5 s

Fig. S4. The gel time of hydrogels with different solid contents.

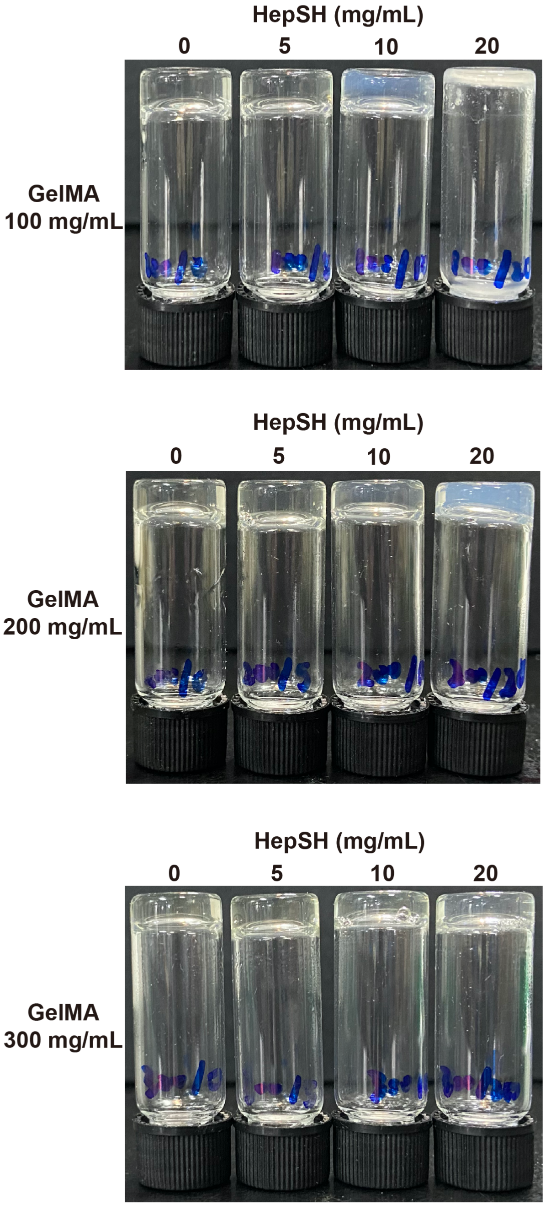

Fig. S5. Photographs of photocured hydrogels with different solid contents.

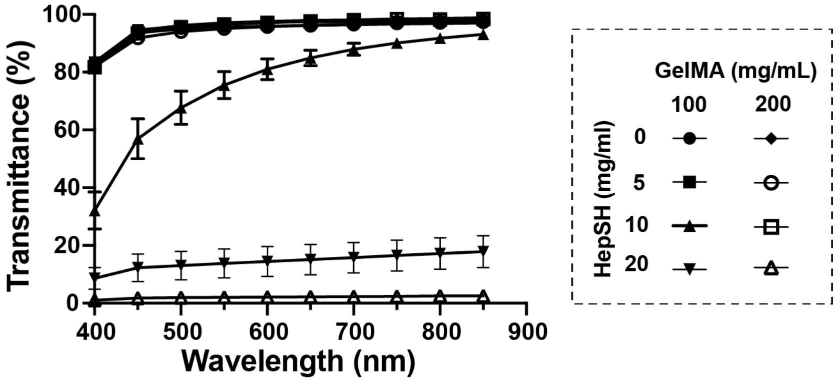

Fig. S6. The transmittance of various Hep@Gel hydrogels in the wavelength range of visible light ($n = 3$).

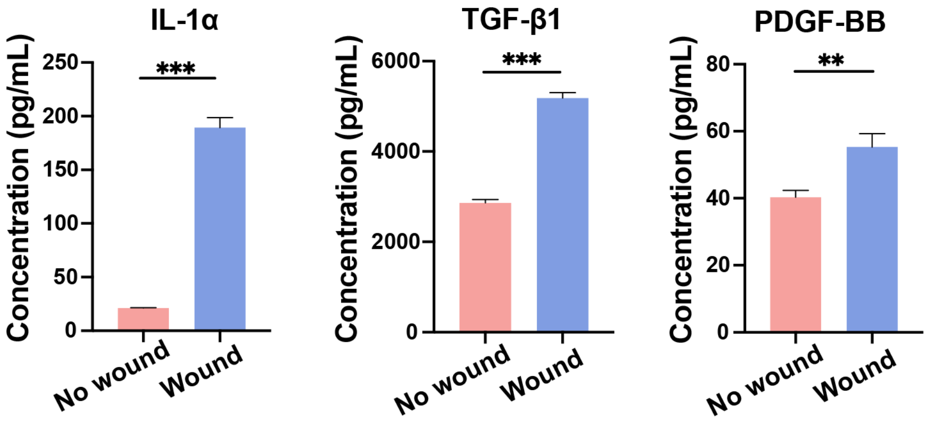

Fig. S7. Quantification of secreted inflammatory and fibrotic cytokines from injured HCECs ($n = 3$, Student's t-test, ** $P < 0.01$, *** $P < 0.001$).

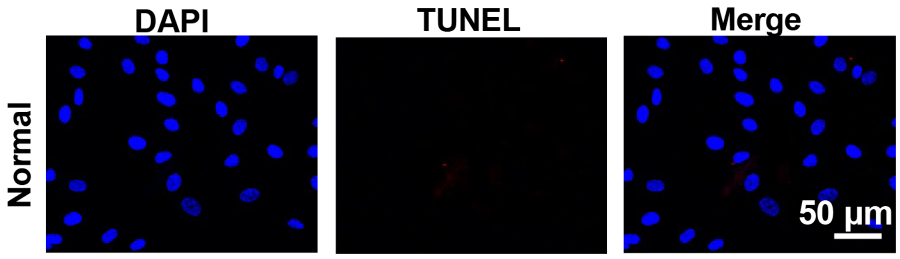

Fig. S8. The representative images of TUNEL staining of untreated keratocytes.

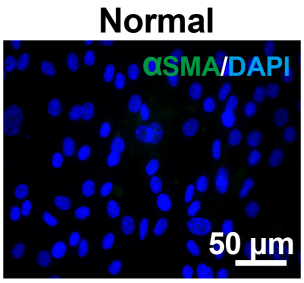

Fig. S9. The representative images of αSMA staining of untreated keratocytes.

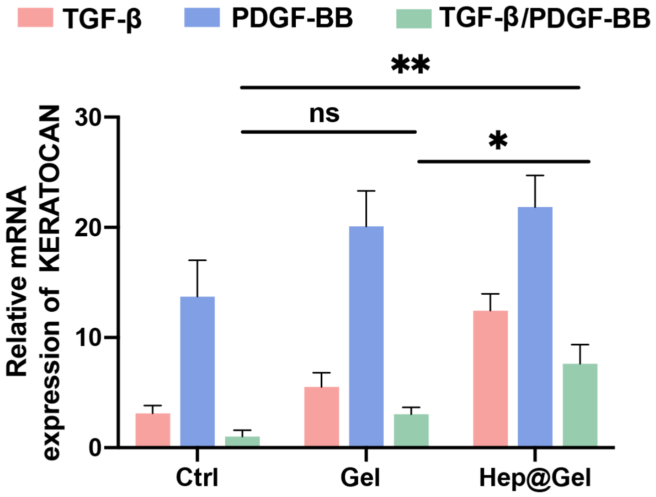

Fig. S10. The mRNA expression of KERATOCAN in keratocytes in the *in vitro* model of corneal injury. KERATOCAN is the keratocyte-specific marker (n = 3, two-way ANOVA test, * $P < 0.05$, ** $P < 0.01$. ns, not significant).

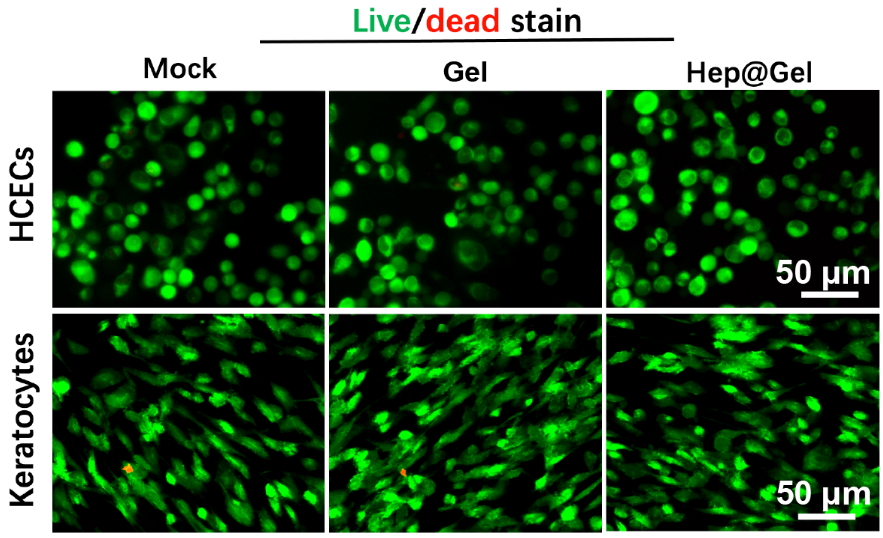

Fig. S11. Live/dead assay of HCECs and human keratocytes seeded on tissue culture well-plate, Gel, or Hep@Gel.

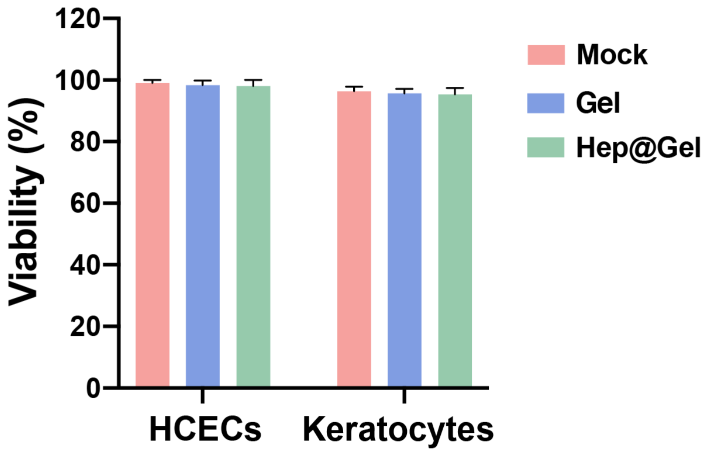

Fig. S12. Viability of HCECs and keratocytes seeded on tissue culture well-plate, Gel, and Hep@Gel according to the live/dead assay (n = 3).

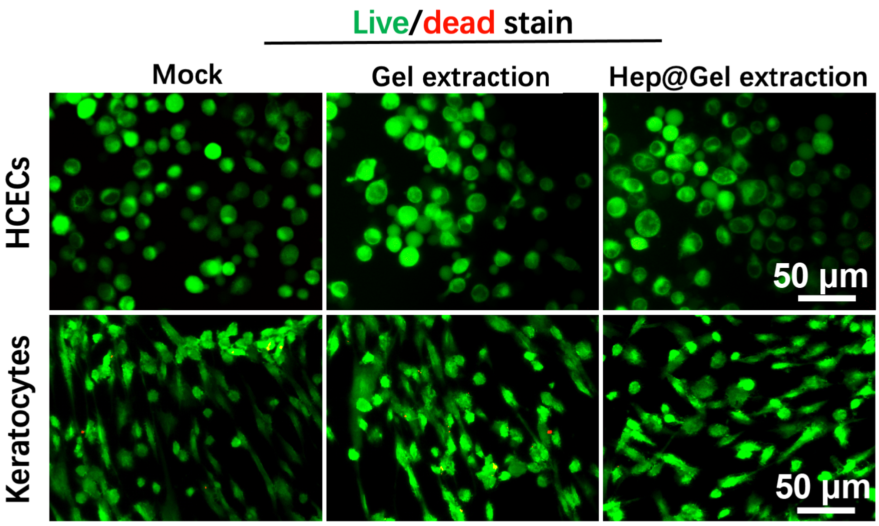

Fig. S13. Live/dead assay of HCECs and human keratocytes cultured in normal medium or hydrogel extraction of Gel and Hep@Gel.

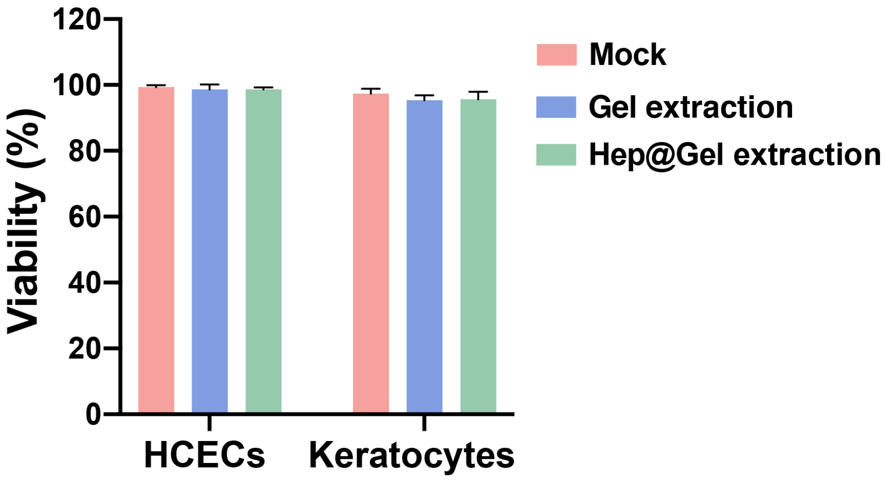

Fig. S14. Viability of HCECs and keratocytes cultured in normal medium or hydrogel extraction of Gel and Hep@Gel according to the live/dead assay (n = 3).

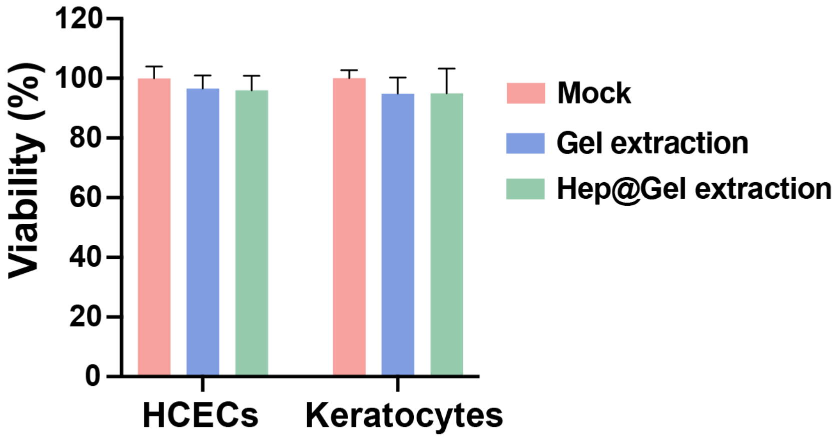

Fig. S15. Viability of HCECs and keratocytes cultured in normal medium or hydrogel extraction according to the CCK-8 assay (*n* = 3).

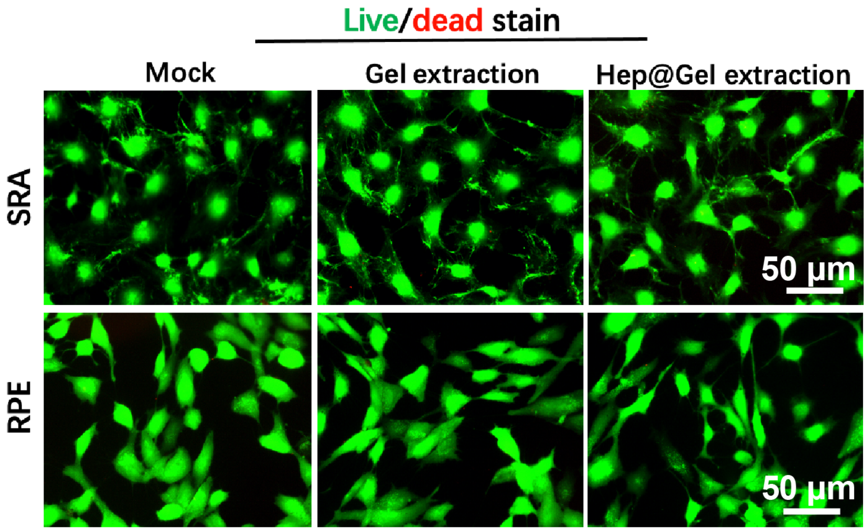

Fig. S16. Live/dead assay of SRA and RPE cultured in normal medium or hydrogel extraction of Gel and Hep@Gel.

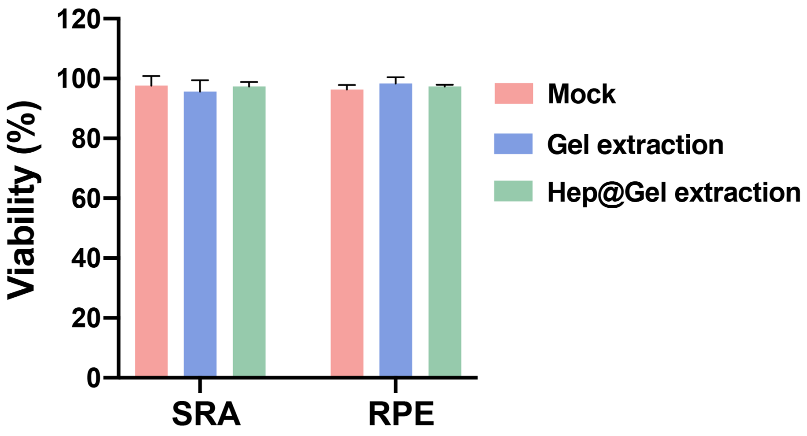

Fig. S17. Viability of SRA and RPE cultured in normal medium or hydrogel extraction of Gel and Hep@Gel according to the live/dead assay (n = 3).

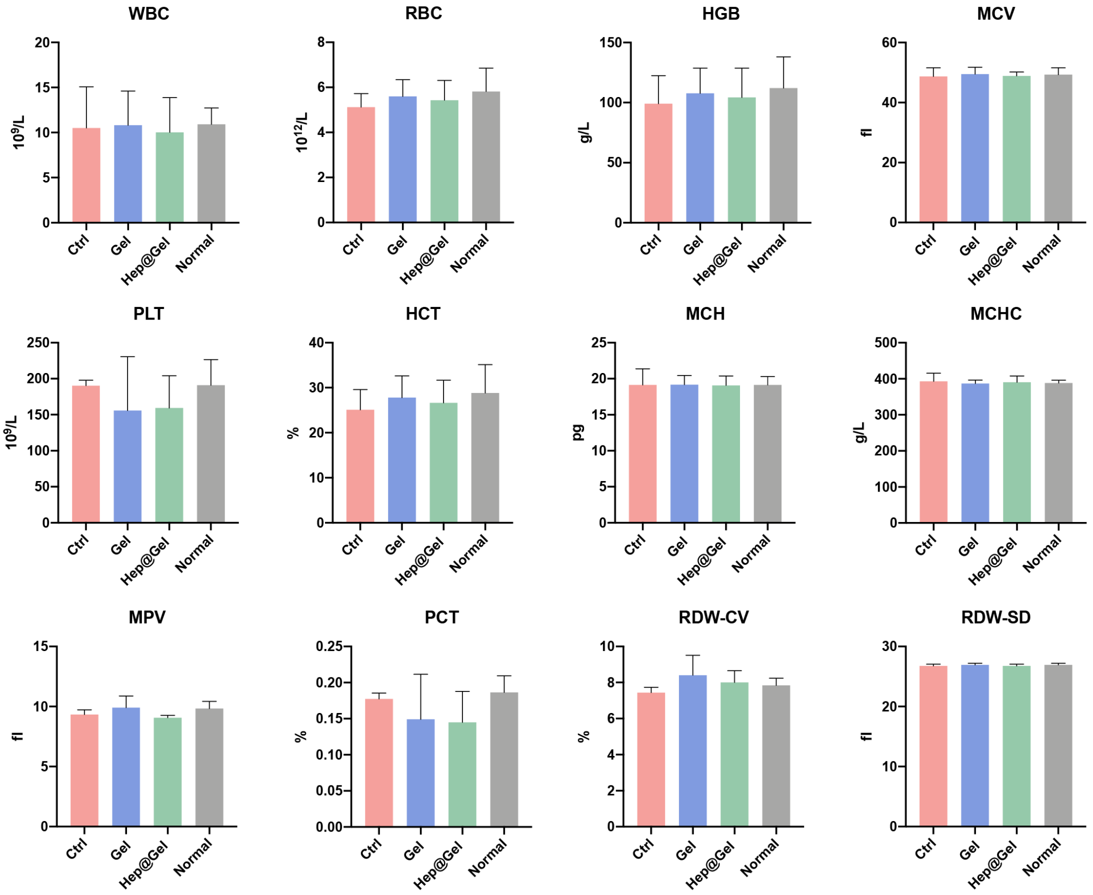

Fig. S18. *In vivo* biocompatibility of Gel and Hep@Gel confirmed by hematology examinations in rabbits (n = 3).

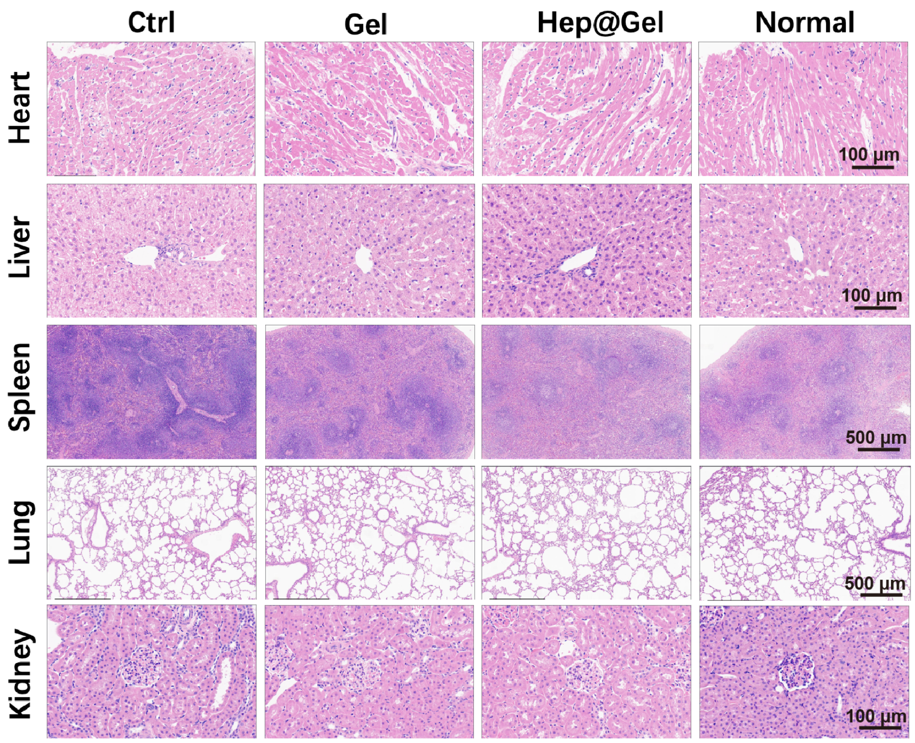

Fig. S19. *In vivo* biocompatibility of Gel and Hep@Gel confirmed by the HE staining of vital organs of rabbits.

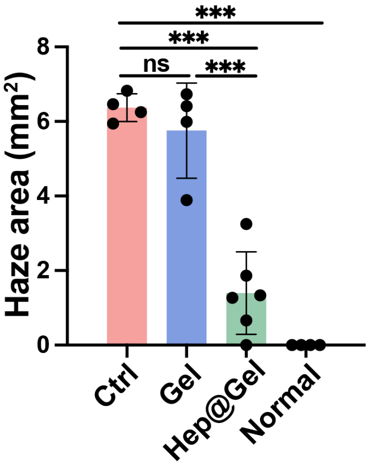

Fig. S20. Quantification of rabbit corneal haze area by slit lamp examination on day 28 post-wounding (n = 4 for the Ctrl, Gel, and Normal groups, and n = 6 for the Hep@Gel group. One-way ANOVA test, *** $P < 0.001$. ns, not significant).

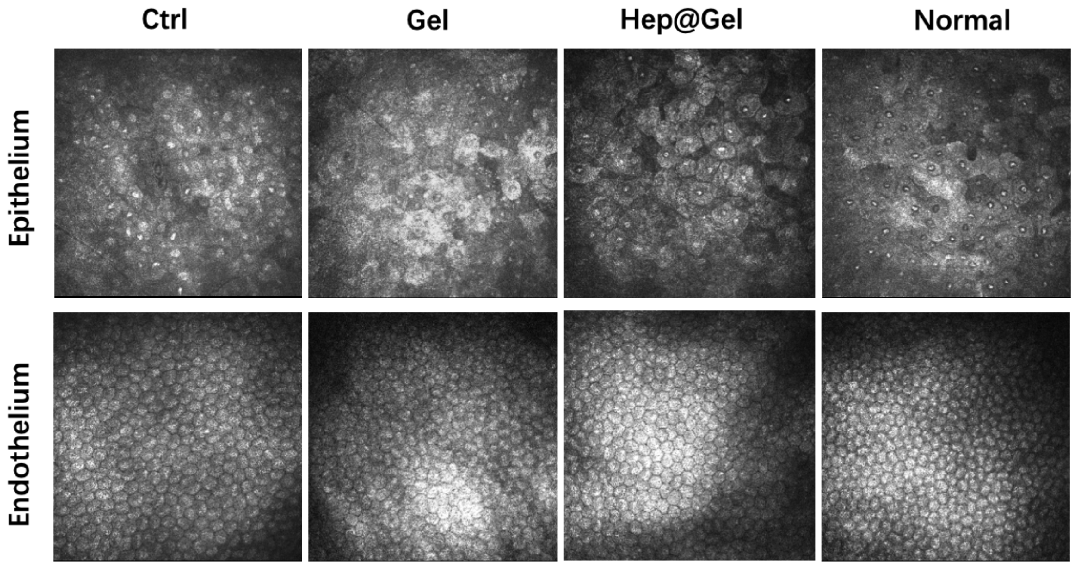

Fig. S21. Confocal microscopy of rabbit corneal epithelium and endothelium on day 28 post-wounding.

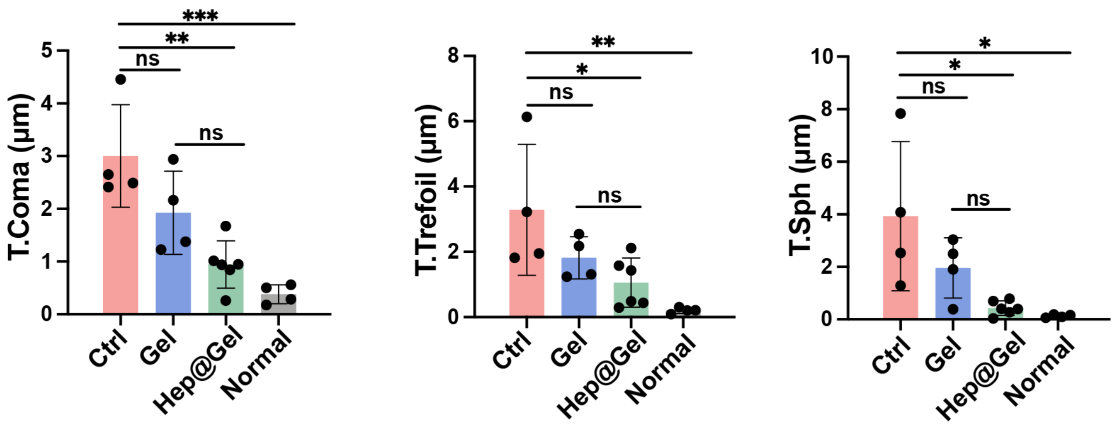

Fig. S22. Wavefront aberration of rabbit corneas on day 28 post-wounding according to the OPD-scan results (n = 4 for the Ctrl, Gel, and Normal groups, and n = 6 for the Hep@Gel group. One-way ANOVA test, * $P < 0.05$, ** $P < 0.01$, *** $P < 0.001$. ns, not significant).

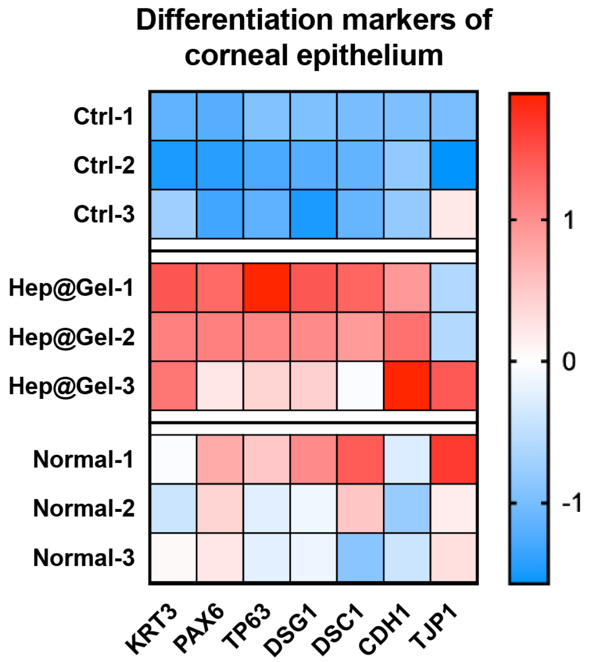

Fig. S23. Heatmap of genes related to the differentiation markers of corneal epithelium in the rabbit corneal defect model.

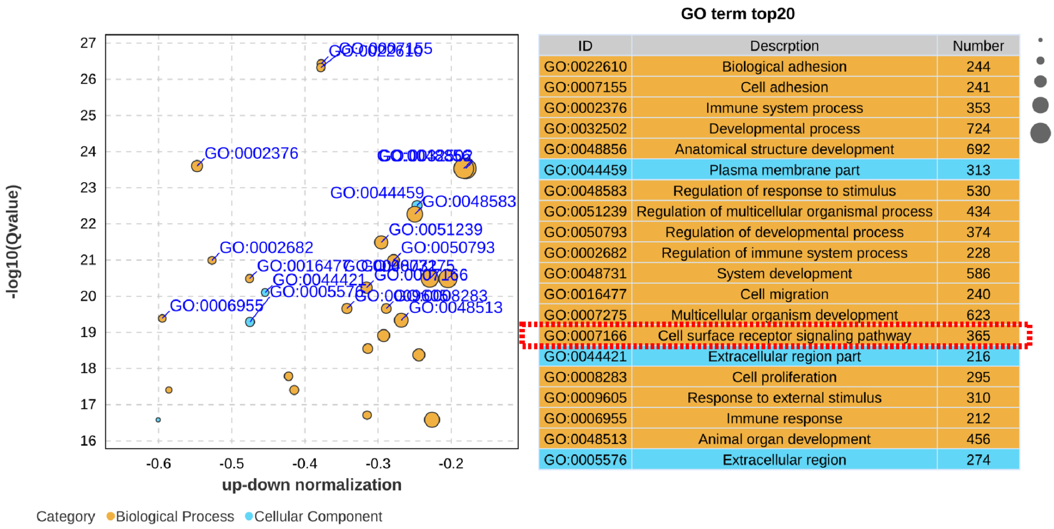

ID	Descrption	Number
GO:0022610	Biological adhesion	244
GO:0007155	Cell adhesion	241
GO:0002376	Immune system process	353
GO:0032502	Developmental process	724
GO:0048856	Anatomical structure development	692
GO:0044459	Plasma membrane part	313
GO:0048583	Regulation of response to stimulus	530
GO:0051239	Regulation of multicellular organismal process	434
GO:0050793	Regulation of developmental process	374
GO:0002682	Regulation of immune system process	228
GO:0048731	System development	586
GO:0016477	Cell migration	240
GO:0007275	Multicellular organism development	623
GO:0007166	Cell surface receptor signaling pathway	365
GO:0044421	Extracellular region part	216
GO:0008283	Cell proliferation	295
GO:0009605	Response to external stimulus	310
GO:0006955	Immune response	212
GO:0048513	Animal organ development	456
GO:0005576	Extracellular region	274

Fig. S24. The top 20 enriched GO pathways between the Ctrl group and the Hep@Gel group in the rabbit corneal defect model.

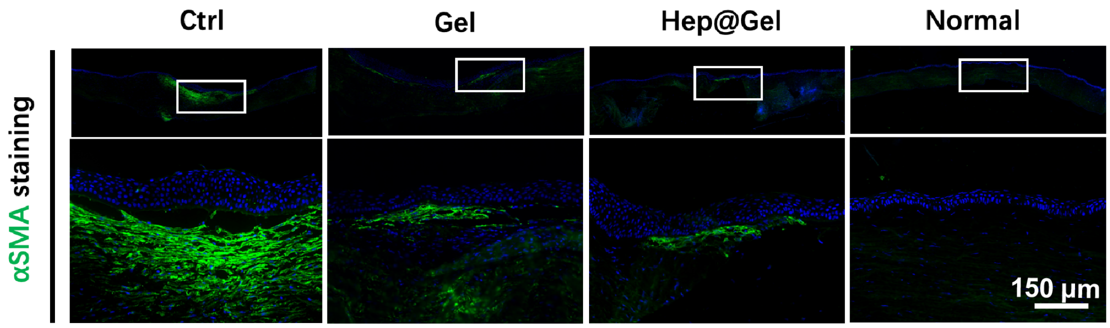

Fig. S25. Representative images of αSMA expression in rabbit corneas on day 28 post-wounding.

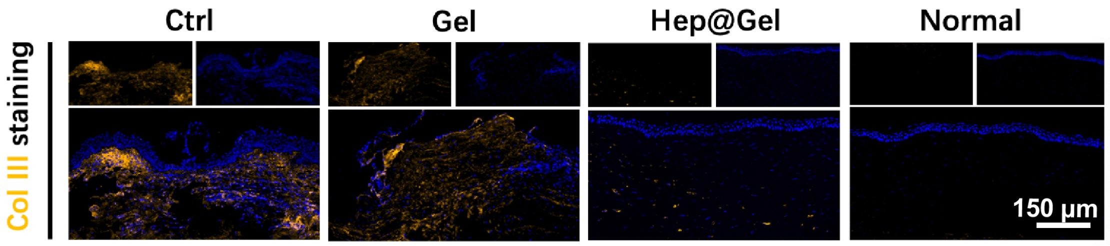

Fig. S26. Representative images of COL III expression in rabbit corneas on day 28 post-wounding.

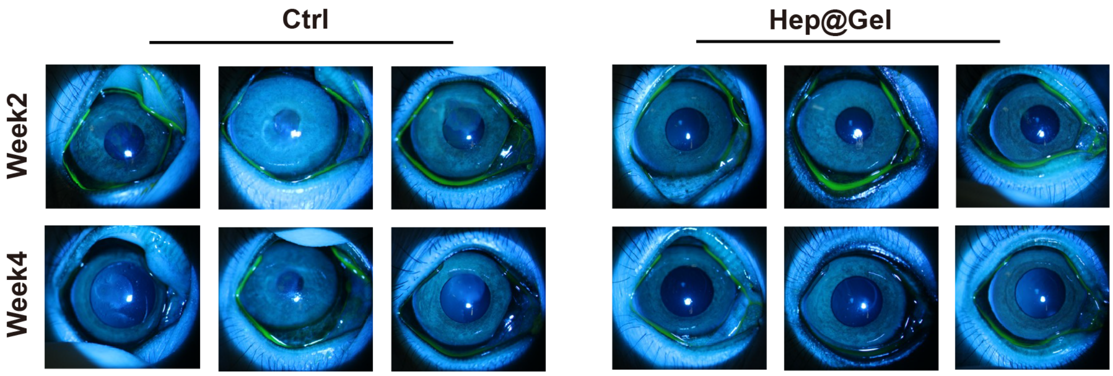

Fig. S27. Evaluation of corneal wound healing by fluorescein staining in week 2 and week 4 post-wounding in the corneal defect model of cynomolgus monkeys.

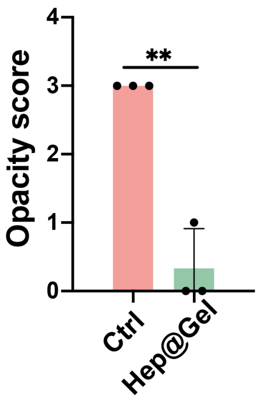

Fig. S28. Quantification of corneal opacity of cynomolgus monkeys by opacity score in week 6 post-wounding. A higher opacity score indicated more severe opacity (n = 3, Student's t-test, ** $P < 0.01$).

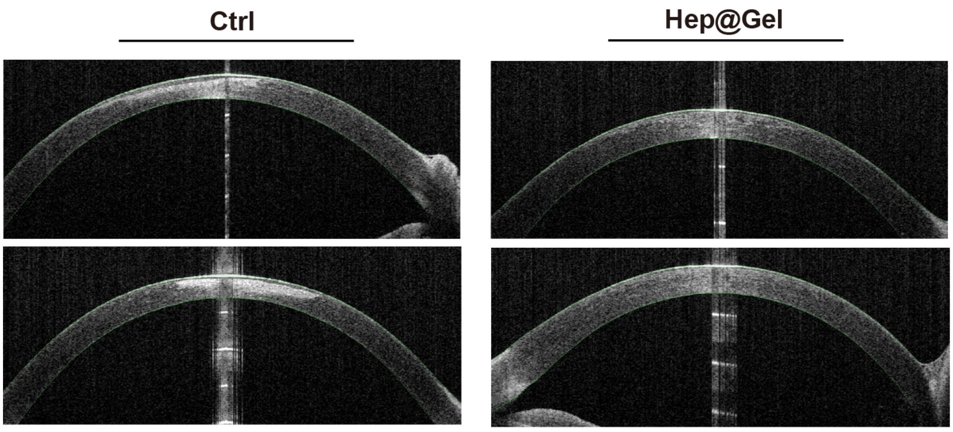

Fig. S29. AS-OCT images of corneas (the other corneas of cynomolgus monkeys not displayed in Fig. 8) in week 6 post-wounding.

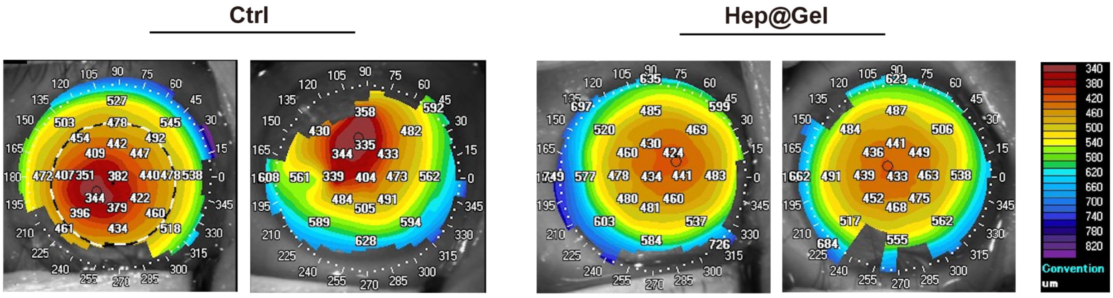

Fig. S30. Corneal pachymetry (the other corneas of cynomolgus monkeys not

displayed in Fig. 8) in week 6 post-wounding.

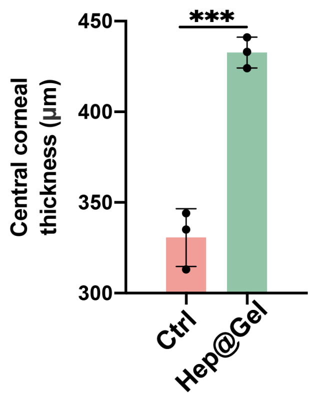

Fig. S31. The quantification of the central corneal thickness of the wound area in cynomolgus monkeys (n = 3, Student's t-test, *** $P < 0.001$).

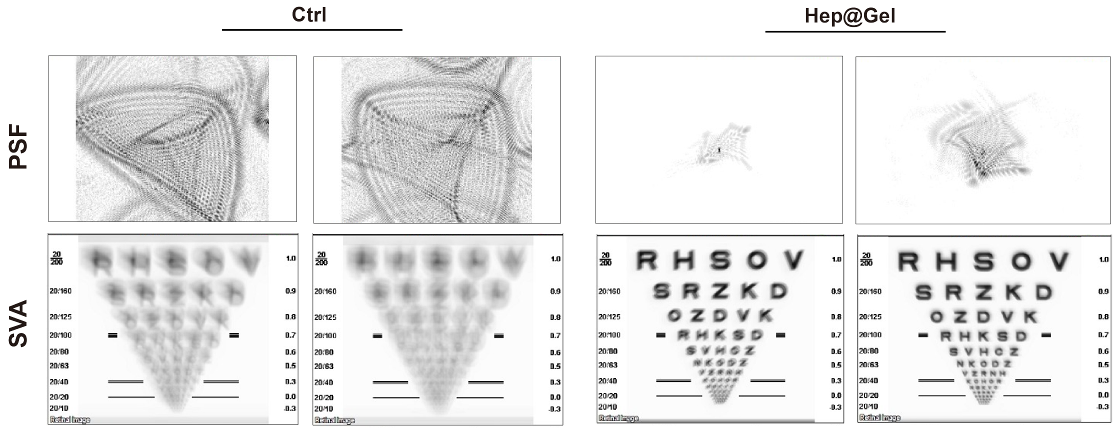

Fig. S32. OPD-scan of corneas (the other corneas of cynomolgus monkeys not displayed in Fig. 8) in week 6 post-wounding.

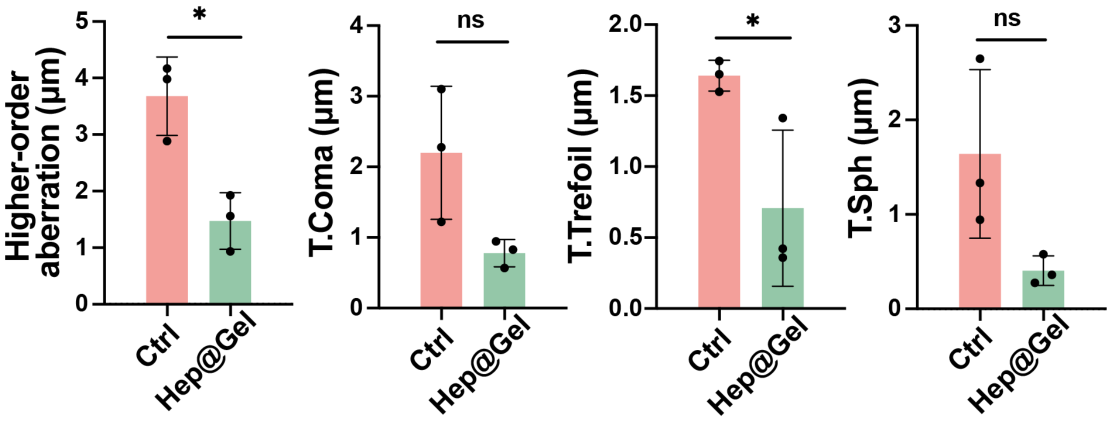

Fig. S33. The corresponding wavefront aberration of the corneas of cynomolgus monkey in week 6 post-wounding (n = 3, Student's t-test, * $P < 0.05$. ns, not significant).

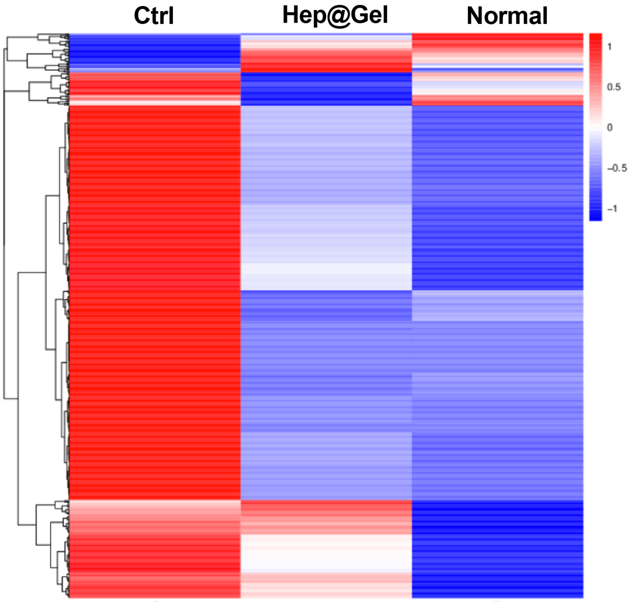

Fig. S34. The heatmap of the differentially expressed proteins between the Ctrl group, the Hep@Gel group, and the Normal group in the corneal defect model of cynomolgus monkeys.

Supplementary tables

Table S1. Classification of proteins in the PPI network.

Function description	Protein		
Translation, ribosomal structure, and biogenesis	P61270	A0A7N9CKL2	A0A2K5TK27
	A0A2K5VCF2	G7PYS4	A0A2K5UEA8
	A0A8J8XC80	I7GML3	A0A2K5WE82
	G7PCA3	A0A2K5V0N6	G7PAK4
	G7NXU7		
Posttranslational modification, protein turnover, chaperones	A0A2K5VUH9	A0A7N9IB42	A0A2K5VR37
	A0A2K5WKL9	A0A2K5VJX7	A0A7N9CKK3
	G7PGF9	A0A2K5V8G9	A0A7N9D2Y5
	A0A2K5W3V7	A0A2K5WSD2	I7GKX3
	Q4R6V2	A0A7N9DA15	A0A2K5X958
	Q4R3H2	A0A2K5TSQ6	
Intracellular trafficking, secretion, and vesicular transport	A0A2K5USY9	G7NWD0	G7PQN7
Lipid transport and metabolism	I7G2W9	A0A2K5V3T1	A0A7N9IF77
Signal transduction mechanisms	A0A2K5VEN4	A0A2K5WQ22	
Mineral absorption (transferrin)	I7GDN5		

Table S2. Primary antibodies for ICC (immunocytochemistry) and IHC (immunohistochemistry).

Antibody	Company	Category number	Dilution	Application
Anti-Collagen IV	Abcam	ab236640	1:300	IHC
Anti-HSPG2	Abcam	ab2501	1:200	IHC
Anti-KSPG	Santa cruz	sc-73518	1:50	IHC
Anti-DSPG2	Proteintech	66847-1-Ig	1:400	IHC
Anti-CSPG1	Proteintech	68350-1-Ig	1:400	IHC
Anti-IL-1	Abcam	ab300501	1:200	IHC
Anti-F4/80	Invitrogen	53-4801-82	1:200	IHC
Anti-MMP9	Abcam	ab76003	1:200	IHC
Anti-αSMA	Proteintech	14395-1-AP	1:400	ICC/IHC
Anti-TGF-β	Abcam	ab190503	1:200	IHC
Anti-PDGF-BB	Abcam	ab23914	1:200	IHC
Anti-Collagen III	Abcam	ab6310	1:200	IHC

Table S3. Primers used in quantitative qPCR.

Species	Gene	Forward Primer (5' -> 3')	Reverse Primer (5' -> 3')
	αSMA	GTGTTGCCCCTGAAGAGCAT	GCTGGGACATTGAAAGTCTCA
Human	KERATOCAN	ACTCACAAAGGTTCCCCGAAT	GGCTGGGACATATTACAGAGACA
	GAPDH	CATTGCCCTCAACGACCACTTTGT	TCTCTCTCTTCCTCTTGTGCTCTTGC

Supplementary movies

Movie S1. The demo video of the burst pressure experiment.
